# Lipid droplet size profiling in yeast

**DOI:** 10.1242/bio.062429

**Published:** 2026-07-09

**Authors:** Katharina Gritsch, Kiara Skrobar, Kristian Bredies, Heimo Wolinski

**Affiliations:** ^1^ Institute of Molecular Biosciences, University of Graz, 8010 Graz, Austria; ^2^ Department of Mathematics and Scientific Computing, University of Graz, 8010 Graz, Austria; ^3^ Field of Excellence BioHealth, University of Graz, 8010 Graz, Austria

**Keywords:** Lipid droplets, Seeding, Deconvolution, Machine learning, Seipin, Yeast

## Abstract

Lipid droplets are central hubs of cellular lipid metabolism and play crucial roles in health and disease. The yeast *Saccharomyces cerevisiae* serves as a powerful model for studying lipid droplet biology at the cellular level. Estimation of the number and size distribution of lipid droplets in a cell population is critical for functional studies. However, accurate segmentation and quantification of lipid droplets through image-based methods present significant challenges. Organelle motion, point-spread function (PSF) overlap of closely associated organelles and heterogeneous fluorescence labeling often compromise histogram-, shape- or machine-learning-based methods. Here, we present an alternative, seeding-based radial raytracing approach that is independent of image histograms and considers the PSF-blurred nature of imaged organelles. To improve lipid droplet resolution and contrast, we applied custom 3D deconvolution and integrated a supervised machine-learning step that corrected typical deconvolution artifacts. An integrated, extensible toolset including deep-learning-based cell registration enables detailed lipid droplet statistics, including user-defined size classes. As a proof of principle, we applied our approach to analyze lipid droplets in wild-type and mutant cells lacking the Sei1-Ldb16 seipin complex required for regular lipid droplet biogenesis.

## Teaser

**Summary:** We present a modular experimental image-analysis platform for yeast lipid droplet quantification that integrates optimized sample preparation, advanced segmentation, size-class analysis, and systematic evaluation of key limitations in organelle morphometry.

## INTRODUCTION

Lipid droplets (LDs) are universal storage organelles for neutral lipids (NLs), mainly triacylglycerol (TAG; fat) and steryl esters (SEs), present in virtually all eukaryotic cells. These dynamic, spherical compartments form at the endoplasmic reticulum (ER) and are enclosed by a phospholipid (PL) monolayer containing fat storage and degradation proteins (Walther et al., 2017). Through organelle contact sites, LDs fulfill metabolic and regulatory functions (Barbosa et al., 2015), while distinct LD subpopulations indicate functional differentiation and broaden metabolic roles (Henne, 2024). Impaired LD turnover is linked to type II diabetes, hepatic steatosis, liver diseases, obesity, and lipodystrophies (Welte, 2015; Walther and Farese, 2012). Abnormal LD metabolism alters LD number and size distribution; therefore, LD quantification is critical for analyses in genetically modified yeast or drug-treated cells. However, quantitative LD analysis remains challenging because sample preparation, image acquisition, and informatics workflows must sufficiently extract LDs from microscopy images to enable quantitative single-cell analysis.

Cytosolic LDs of yeast are highly mobile, show oscillating lateral and axial movements, and are physically inherited by daughter cells (Wolinski et al., 2011). These dynamics complicate LD imaging with scanning systems such as confocal laser scanning microscopy because repeated spatial acquisition of the same organelle may occur. Chemical fixation can immobilize LDs before imaging but may also disintegrate LDs or cause fusion of adjacent organelles (DiDonato and Brasaemle, 2003). Thus, optimized fixation is required to immobilize LDs during acquisition while preserving LD morphology.

Yeast cells are approximately 5 μm in size, whereas LD dimensions partly lie below the optical resolution limit. In addition, LDs can be closely associated, forming ‘pearl-like’ arrangements, as observed in wild-type (WT) cells under starvation conditions (Barbosa and Siniossoglou, 2016). Therefore, optimized measurement settings and maximum optical resolution are necessary for sufficient organelle acquisition. Various methods are used to label and detect LDs at the cellular level. Advanced techniques such as coherent anti-Stokes Raman scattering microscopy allow label-free LD detection in different cell systems, including yeast (Wolinski et al., 2012; Radulovic et al., 2013). However, LDs are typically labeled with GFP-tagged proteins, fluorescent fatty-acid analogs, or organelle-specific dyes such as BODIPY 493/503 and are optimally imaged by high-resolution fluorescence microscopy, including confocal laser scanning microscopy (Wolinski and Kohlwein, 2014).

As with any optical system, the acquired 3D image of fluorescently labeled LDs represents the convolution of the object with the system-specific point-spread function (PSF), rather than its original shape. Image restoration methods such as iterative Richardson-Lucy (RL) deconvolution (Richardson, 1972) can partly compensate for this effect but require information about the imaging system PSF (Swedlow, 2013). Deconvolution algorithms are implemented in commercial systems, open-source software (Sage et al., 2017), and Python libraries or packages (van der Walt et al., 2014; Perdigão et al., 2024) and can improve LD resolution in volumetric images, including high-speed confocal data from our laboratory (Pribasnig et al., 2018). However, parameters must be carefully selected to avoid noise amplification, ringing at high-contrast object borders (Sage et al., 2017), or PSF mismatch artifacts (von Tiedemann et al., 2006). Artifact mitigation, including regularization (Dey et al., 2006; Sage et al., 2017), can be limited when datasets combine very small, dim LDs with large, high-contrast objects showing flattened intensity regions. Machine-learning approaches such as random-forest pixel classification (Berg et al., 2019) may help segment user-trained artifactual structures and enable adaptive filtering of partially artificial deconvolution data.

For quantification, fluorescently labeled LDs must be segmented using methods available in Fiji (Schindelin et al., 2012), Icy (de Chaumont et al., 2012), CellProfiler (Lamprecht et al., 2007), and related tools. Besides edge-detection and machine-learning approaches (Exner et al., 2019; Princová et al., 2019; Adomshick et al., 2020; Dejgaard and Presley, 2014; Varinli et al., 2015; McDonough et al., 2009; Speer et al., 2024), histogram-based methods such as Otsu (Otsu, 1979) and Huang (Huang and Wang, 1995) are commonly applied. Otsu maximizes interclass variance assuming bimodality, whereas Huang minimizes class-assignment fuzziness without requiring a bimodal histogram. Nevertheless, both depend on intensity histograms and are affected by fluorescence labeling variability, photon-detection settings, and intensity differences between small and large LDs, reducing performance and comparability. In contrast, a seeding-based ‘radial raytracing’ (SRR) approach, as presented in this work, that identifies seeded objects using criteria largely independent of absolute gray values and global histograms, while partially considering PSF-affected object shape, is advantageous for LD registration.

Single-cell analysis additionally requires cell-boundary or cell-area detection, using spectrally compatible fluorescent fusion proteins or cytosolic reference dyes (Chong et al., 2015). Alternatively, acquired bright-field or differential-interference contrast images of yeast cells can be used for cell detection including deep-learning approaches (Wolinski et al., 2009; Dietler et al., 2020; Lu et al., 2019; Bredies and Wolinski, 2011). This strategy has the advantage that no additional fluorescent co-labeling of the cell border or body is required.

Loss-of-function mutations in seipin, a protein encoded by the BerardinelliSeip congenital lipodystrophy type 2 gene (*BSCL2*), are associated with congenital generalized lipodystrophy type 2 (CGL-type 2), the most severe form of genetic lipodystrophy in humans. CGL-type 2 results in impaired adipogenesis and a near-complete absence of adipose tissue. Seipin is essential for normal LD biogenesis (Salo, 2023; Klug et al., 2021) but also plays an important role in PL homeostasis (Pagac et al., 2016; Yan et al., 2018), although the molecular mechanisms underlying *BSCL2* pathogenesis remain elusive. In yeast, seipin is a complex composed of two proteins, Sei1 and Ldb16 (Wang et al., 2014), which work together with accessory proteins (Wang et al., 2024; Bohnert, 2020). At the cellular level, deletion of either *SEI1* or *LDB16* as well as the double deletion of these genes results in striking growth-medium-dependent abnormal LD morphology, such as supersized and/or clusters of smaller LDs. These aberrant LD phenotypes demonstrate the critical role of seipin in regular LD formation and maintenance within the yeast model as well (Szymanski et al., 2007; Fei et al., 2008; Cartwright et al., 2014; Wolinski et al., 2015; Grippa et al., 2015; Romanauska and Köhler, 2021). Here, we applied our approach for quantitative, comparative analysis of LDs in WT cells and the *sei1*Δ *ldb16*Δ double-deletion mutant.

In summary, we present a comprehensive experimental toolset encompassing sample preparation, 3D deconvolution, machine-learning-assisted region-specific data filtering, SRR-based LD detection, and deep-learning yeast cell registration, enabling statistical analysis of LD number and size-class distribution at the single-cell level. Despite these advances, we discuss the remaining limitations of image-based LD quantification and outline avenues for future improvement.

## RESULTS

The modular and extensible image-informatics pipeline [‘Lipid Droplet (LD)-Toolbox’] comprises different steps, utilizing mainly newly developed Python scripts (‘modules’) and existing open-source software. The workflow and involved modules and tools to quantify LDs in yeast cell populations are shown in Fig. S1. A summary and description of created modules are shown in Table S1. For user-friendly processing of the pipeline, a workflow and file manager are provided, which also include a help function with the description of the parameters of each module. Movie 1 shows a demonstration workflow.

### SRR approach for LD registration

Our approach is based on processing of maximum-intensity projections (MIPs) of 3D *z*-stacks of fluorescently labeled LDs. The LD quantification process begins with the initial detection of the LD centers in the projections. From these centers, the LD intensity decay is traced radially until specific stopping criteria are met. In this respect, a 360° radial scan with a user-defined number of virtual rays is conducted (e.g. 25 rays) from the detected LD centers. When a 50% intensity drop is measured in a user-defined minimum number of virtual rays (e.g. a minimum of ten rays), the scanning process stops, and the LDs are registered and described by circles. This SRR also allows for the detection of closely associated LDs for which intensity profiles partially overlap. Fig. S2 illustrates the basic principle of the LD registration approach.

### Compensation for LD movement

LDs show fast subcellular movements, particularly in actively growing yeast cells (Wolinski et al., 2011). We also observed oscillating movements of LDs in stationary-phase cultures (Movie 2). To immobilize LDs and prevent a ‘motion’ effect and multiple acquisitions of the same structures, we include a short formaldehyde fixation step before imaging. This treatment immobilizes LDs without artificial fusion of closely associated organelles. However, under starvation conditions, some fluorescently labeled structures were also detected in vacuoles, which could not be fixed without LD fusion artifacts. To identify residual movements in fixed cells, we applied a routine to detect abrupt contrast changes between consecutive slices within individual *z*-stacks and to segment such dynamic LDs (‘LD_3D_MovementFinder’ module). The resulting binary data was then used to refine the LD detection process in a later stage of the workflow (Fig. S3, Movie 3).

### Resolution and contrast enhancement of LDs using open-source 3D deconvolution

**Fig. 1.**
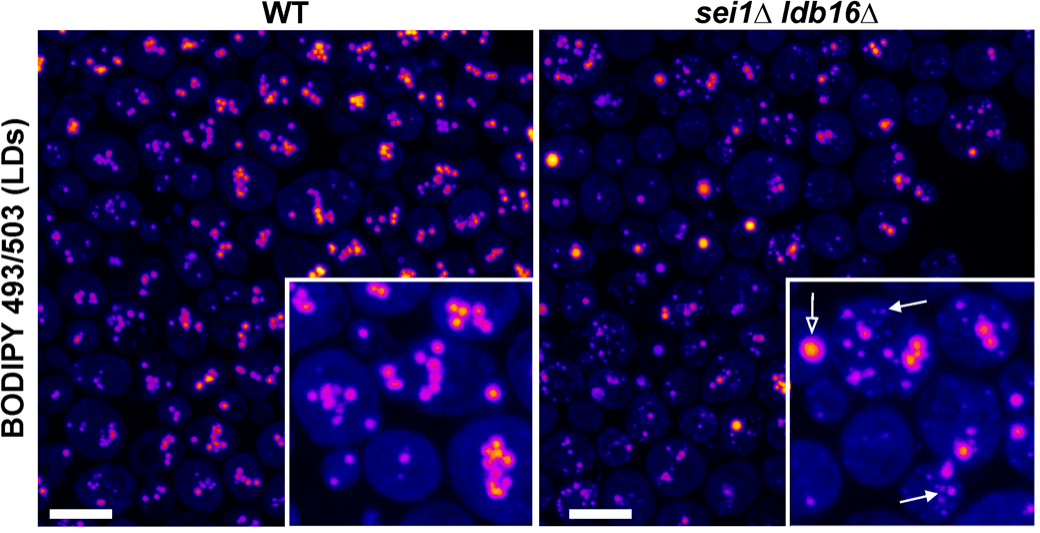
**LD phenotypes in WT and *sei1*Δ *ldb16*Δ cell cultivated for 48 h in rich medium.** Distribution of LD sizes in WT cells without observable extreme size differences (left image). Highly heterogeneous LD sizes in the double-deletion mutant. In contrast to the WT, the double-deletion mutant shows a fraction of very large (open arrow) and very small LDs in the cells (closed arrows). Insets represent enlarged regions of the datasets. Gray-level intensity of insets was increased for display. Images represent MIPs of raw *z*-stacks acquired from BODIPY-493/503-labeled LDs. Scale bars: 5 μm.

We initially inspected raw data from WT and *sei1*Δ *ldb16*Δ cells. WT cells showed concentrated cytosolic LDs, previously associated with the nucleus and nuclear vacuole junctions under starvation conditions (Barbosa and Siniossoglou, 2016). The WT LD size appeared homogeneous; no extreme differences within the organelle population were visible. In contrast, *sei1*Δ *ldb16*Δ cells showed heterogeneous LD sizes, ranging from very large to partly clustered small LDs, as previously observed in *sei1*Δ cells (Choudhary et al., 2020). In addition, tiny, low-contrast LDs were scattered throughout mutant cells and were apparently less abundant in WT cells (Fig. 1; Fig. S4).

**Fig. 2.**
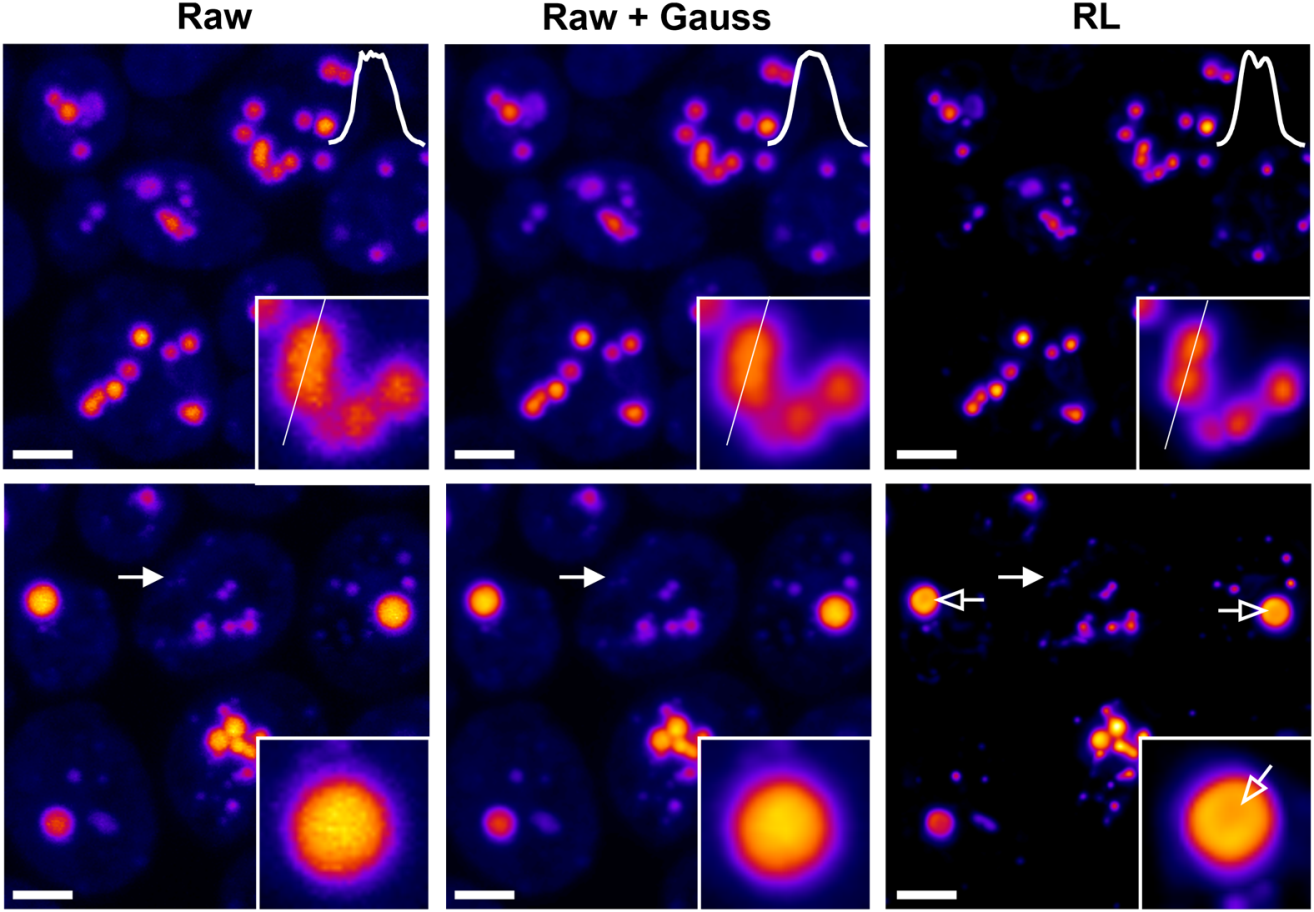
**Effect of open-source deconvolution on resolution and contrast of LDs in WT and *sei1*Δ *ldb16*Δ cells.** Raw data (Raw), 3D Gaussian-filtered data (Raw+Gauss) and deconvolved data (RL) of LDs in WT cells of MIPs of BODIPY-493/503-labeled LDs. The intensity profiles computed for the lines in the inset show two separate peaks in the deconvolution but not in the raw and Gaussian-filtered data (top panel). Limited contrast of very dim LDs in raw and Gaussian-filtered data. Significant enhanced contrast of such LDs upon deconvolution (closed arrows). Introduction of deconvolution artifacts into bright and large LDs reflected by decreased intensity of the center of the organelle (open arrows and RL inset, bottom panel; also shown in Fig. 4). Insets represent enlarged areas of the images. Scale bars: 2 μm.

To increase resolution of closely associated LDs and contrast of very small, dim LDs, we applied RL deconvolution (Lucy, 1974; Richardson, 1972) (‘LD_3D_Deconvolution’ module). Deconvolution requires the system-specific PSF, extracted from eight measured and merged 200 nm fluorescent reference beads. For acquisition, LD *z*-stacks were acquired without line averaging and with increased scan speed. Intensity maxima of closely associated LDs were often unresolved in raw data and after common Gaussian noise reduction; 3D RL deconvolution significantly improved LD resolution, made object centers detectable, and particularly enhanced contrast of very small, dim LDs. However, RL deconvolution introduced artifacts in large, bright LDs with flat central intensity profiles, producing donut-like structures. This artifact occurred mainly in supersized LDs of *sei1*Δ *ldb16*Δ cells and interfered with LD-center detection and registration (Fig. 2).

Nevertheless, RL deconvolution improved image quality for most LDs. The same strategy was validated for spatially distributed subcellular structures, such as the yeast mitochondrial network, which lacks very large, flat intensity regions and better matches the measured PSF. Even noisy image data showed improved quality after deconvolution (Fig. S5).

### Machine-learning-assisted adaptive filtering of partial artificial deconvolution data

**Fig. 3.**
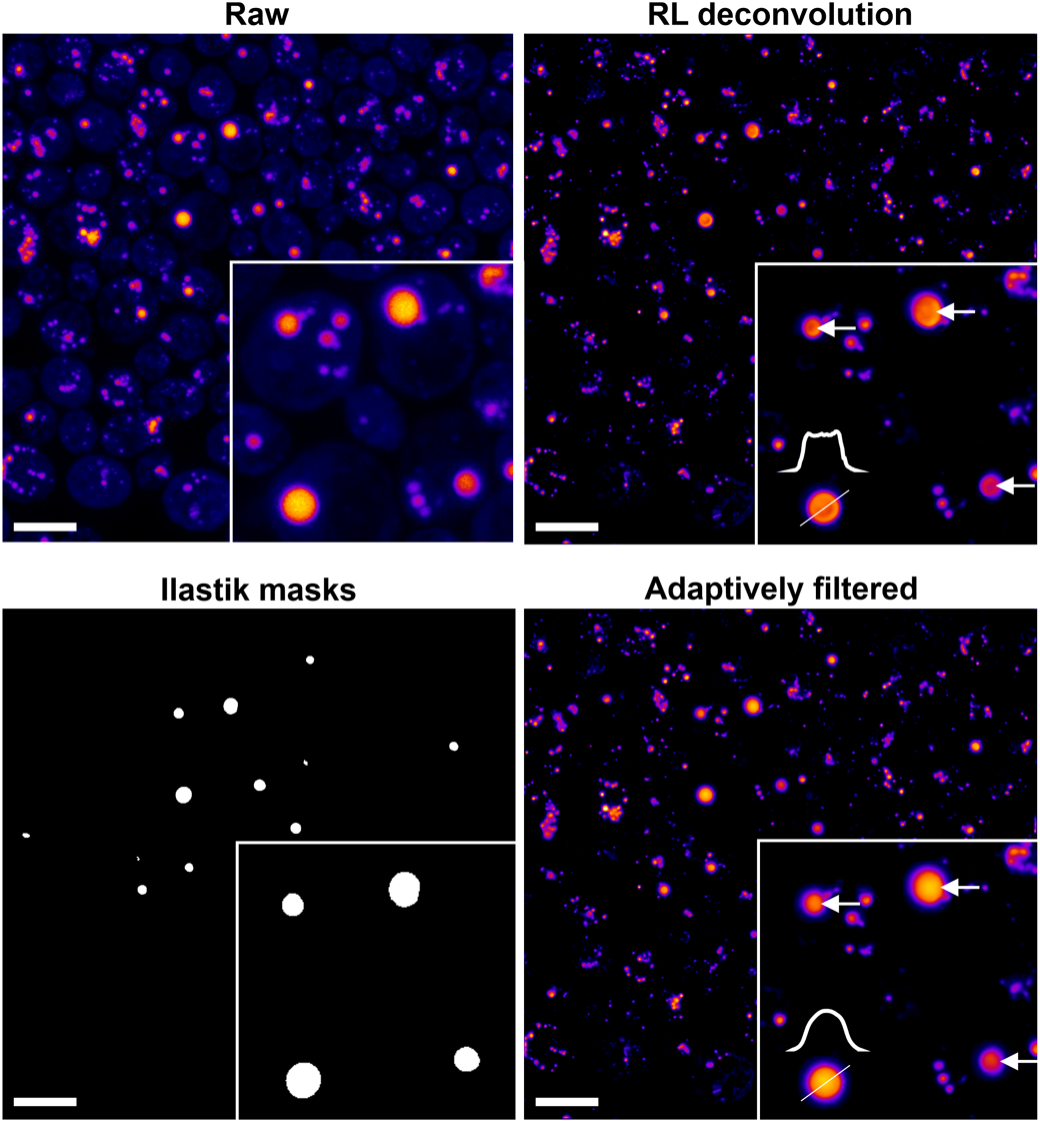
**Machine-learning-assisted adaptive filtering to compensate for deconvolution artifacts in larger LDs occurring in *sei1*Δ *ldb16*Δ cells.** Artifacts introduced into large LDs by RL deconvolution indicated by a decrease in the intensity of LD centers (top panel, RL deconvolution, inset, closed arrows). The intensity profile in the inset represents the plot profile of the line through a large LD. Compensation of the deconvolution artifacts and Gaussian intensity profile of recomputed LDs using masks (bottom panel, ilastik masks) in the adaptively filtered data (bottom panel, adaptively filtered, inset, closed arrows). Non-masked areas remain unaffected. 2D LD masks. Fluorescence images show MIPs of BODIPY-493/503-labeled LDs. Scale bars: 2 μm. Insets represent the same enlarged area of the image. White curves show the fluorescence-intensity profiles along the indicated lines.

To address the deconvolution artifact issue, we applied a machine-learning approach to initially mask LDs showing deconvolution artifacts and abnormal intensity drop in the midst of larger LDs. To create the masks, a training dataset for segmentation of artificially processed LDs was created using the parallel random-forest pixel classifier implemented in ilastik (Berg et al., 2019) and refined using the ‘LD_RingingCleaner’ module. Subsequently, the original pixel distribution in such masked areas was restored, Gaussian filtered (σ=2) and processed with an algorithm to ensure a smooth transition to the outer areas (‘LD_AdaptiveFiltering’ module). As a result, the contrast of small, deconvolved LDs remains significantly improved, while large LDs are unaffected by deconvolution artifacts in the adaptively filtered datasets (Fig. 3). The ‘LD_RingingCleaner’ module also includes a feature to mask artificial LDs in the images manually without ilastik.

### Performance in detection and separation of LDs

To obtain a baseline for the LD intensity detection threshold, we first determined the threshold that correctly measured deconvolved 200 nm fluorescent reference beads with SRR and benchmarked it against histogram-based Otsu and Huang segmentation. With SRR, the mean area corresponding to the 200 nm bead diameter was obtained using 70% intensity decay from the detected bead center. In the same images, Otsu overestimated bead area by ∼46% and Huang by ∼324% (Fig. S6). Because BODIPY 493/503 also labels PL membranes (Wolinski and Kohlwein, 2015) and larger LDs deviate from the mainly PSF-limited bead pattern, BODIPY-stained LDs may appear broader than their actual size. We therefore used a higher radial threshold (50% intensity decay) as a pragmatic compromise to detect very small and larger LDs while limiting diameter overestimation.

**Fig. 4.**
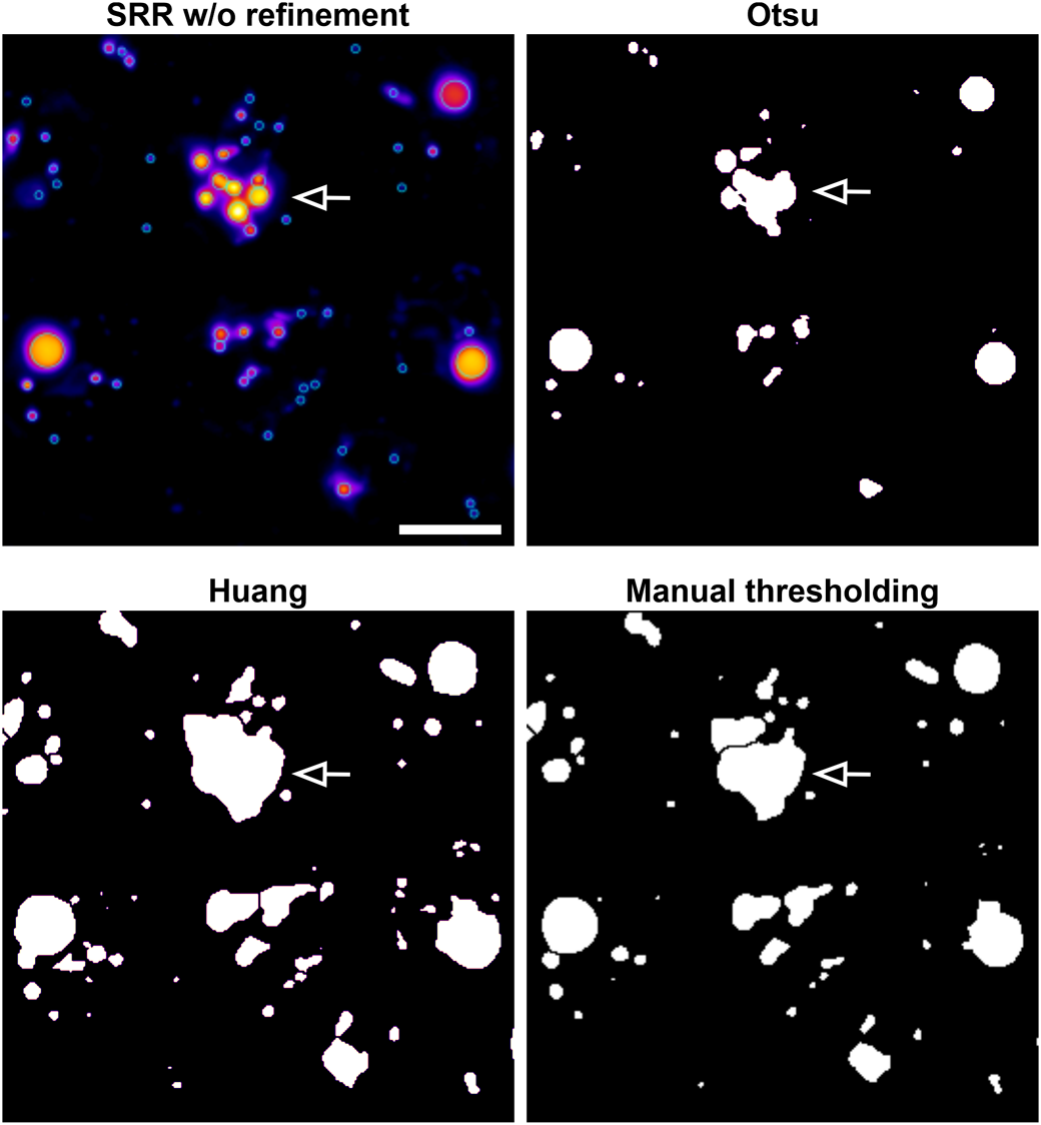
**Comparison of SRR and histogram-based segmentation methods to detect LDs of variable sizes in *sei1*Δ *ldb16*Δ cells.** The SRR LD registration process approximated both closely associated and small LDs (SRR w/o refinement; open arrow). Limitation of Otsu, Huang and manual thresholding to separate closely associated LDs despite the application of the Fiji watershed algorithm (Otsu, Huang, Manual thresholding; open arrows). The fluorescence image represents a cropped, adaptively filtered MIP of the BODIPY-493/503-labeled LDs shown in Fig. 2. Scale bar: 2 μm.

The h-maxima method was used to detect initial local intensity maxima (Soille, 2004). We then applied Density-Based Spatial Clustering of Applications with Noise (DBSCAN) to cluster extremely close local maxima and compute a single core cluster (Ester et al., 1996). Object centers were further optimized based on the MIP of the measured PSF; SRR was then applied from these centers. We evaluated the registration of individual closely associated LDs and very small LDs in adaptively filtered *sei1*Δ *ldb16*Δ data and compared the results with those of the Otsu and Huang methods. In these cases, LDs were additionally separated by a Fiji watershed algorithm (Vincent and Soille, 1991). Otsu separated closely associated LDs only partly and was limited in detecting dimmer, smaller LDs. Huang improved the detection of smaller LDs but failed to separate closely associated LDs and strongly overestimated organelle size, as shown for reference beads (Fig. S6). In contrast, SRR sufficiently detected both very closely associated and small, dim LDs (Fig. 4); SRR-processed data therefore allow LD-size-class analysis.

In practice, recording fluorescently labeled LDs requires photomultiplier adjustment to prevent detector saturation, especially in samples with heterogeneous LD sizes and signal intensities. Fluorescence labeling may also vary between samples and experiments. To assess LD registration across different intensities, we artificially decreased LD image intensity in 10% increments and applied the registration process. The approach was relatively stable across variable gray-level intensities. Although computed LD diameters decreased consistently, deviation remained within ∼12% of the reference when image intensity was reduced to 50% of the original value (Fig. S7).

### Yeast cell registration and LD assignment to cells

**Fig. 5.**
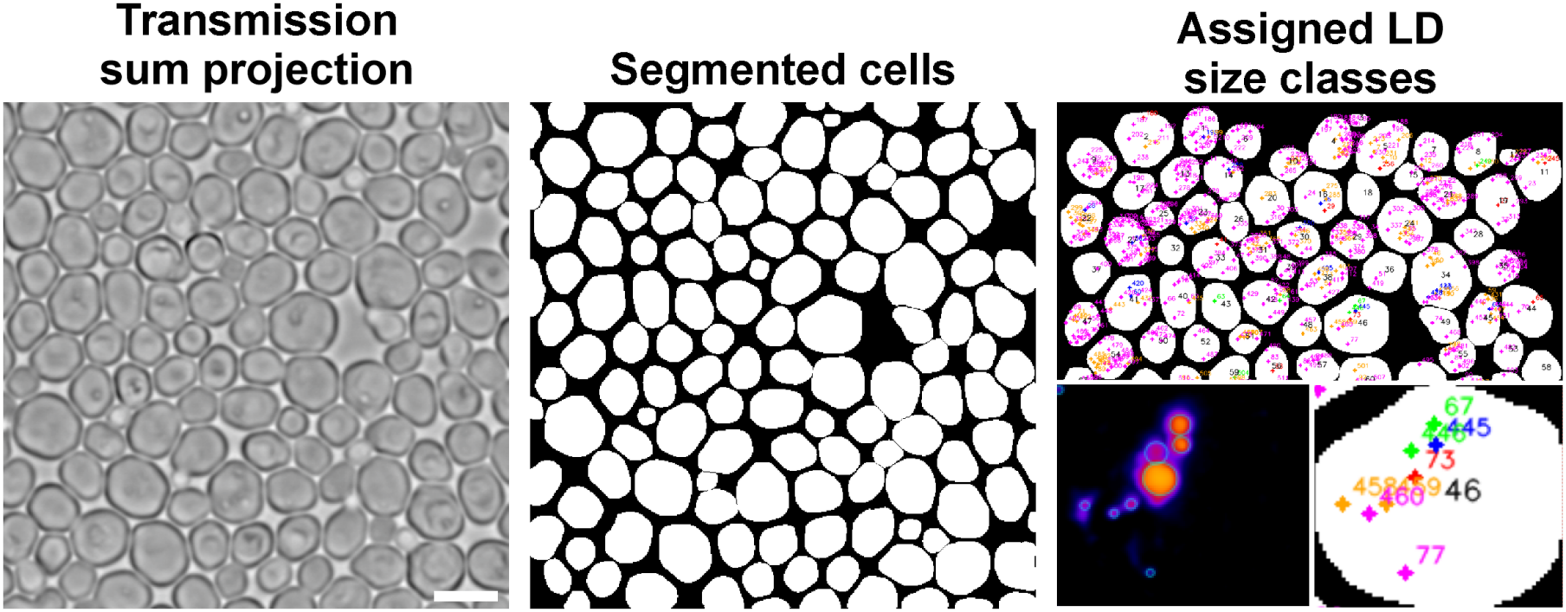
**Deep-learning-assisted detection of yeast cells in transmission images and LD-cell assignment for statistical analyses.** Sum projection of 3D transmission data of *sei1*Δ *ldb16*Δ cells (left image). Segmented cells using ‘YeastSpotter’ (middle image). Refined segmentation result including assigned LD coordinates. User-defined LD-size classes are indicated by different star and text colors (inset, right image). Border cells are automatically excluded from analysis. Insets represent an enlarged area of this image (adaptively filtered MIP of BODIPY-493/503-labeled LDs, left inset; corresponding assigned LD-size classes, right inset). Scale bar: 5 μm.

For statistically relevant analysis of detected LDs in yeast cell populations, cell areas or boundaries must be registered. We used YeastSpotter, a deep-learning method (Lu et al., 2019), to approximate cell bodies in sum projections of simultaneously acquired high-resolution transmission images. YeastSpotter sufficiently segmented yeast areas in these projections. Raw segmentations were refined (‘LD_CellMaskRefiner’ module), and insufficient segmentations, such as false spaces between closely associated cells, were corrected using the interactive ‘LD_CellMaskEditor’ module. Detected LDs were then assigned to individual cells based on computed center coordinates. The ‘LD_CellAssigner’ module allows user-defined LD-size classes and outputs mean LD size, LD number per cell or cell area, and detailed size-distribution statistics across the population (Fig. 5).

### Morphometric analyses of LDs in WT and *sei1*Δ *ldb16*Δ cells

**Fig. 6.**
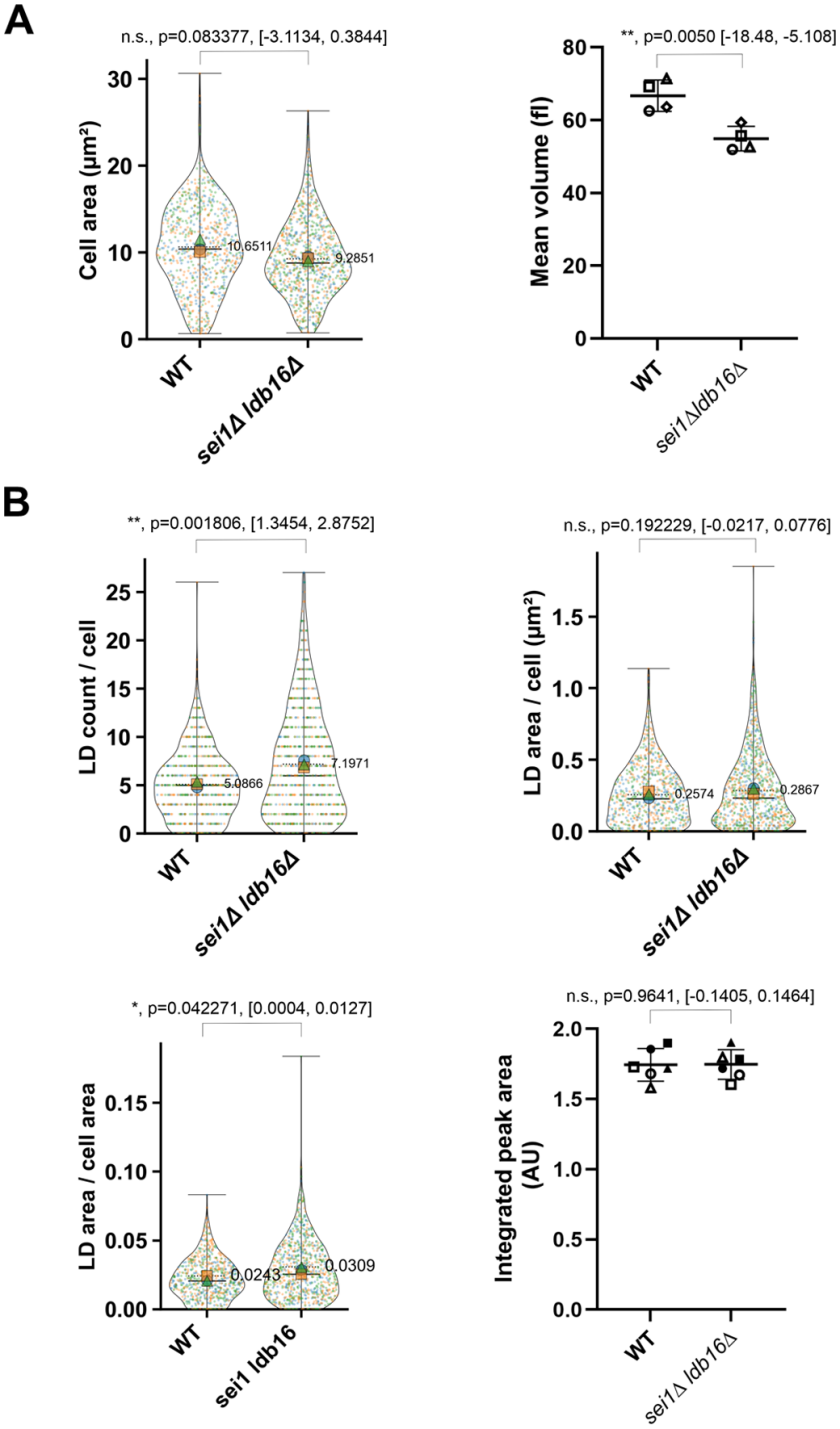
**Image-based quantification of cell size and LD parameters in WT and *sei1*Δ *ldb16*Δ mutant cells.** Quantification of cell areas in *sei1*Δ *ldb16*Δ double-deletion mutants compared to WT (A, right). Measurement of mean cell volume using CASY™ cell counter of four biological replicates. Symbol shapes indicate the replicates (A, left). LD count per cell (B, top panel, left) and LD area per cell (B, top panel, right). Mean LD area computed per cell area (B, bottom panel, left). WT, *n*=764 cells; *sei1*Δ *ldb16*Δ, *n*=942 cells. Violin plots: solid line, median; dashed line, mean; symbols, replicate means; vertical lines, minimum to maximum; colored circles, individual replicate values. Analysis of the NL content of WT and *sei1*Δ *ldb16*Δ double-deletion mutant cells using TLC. The NL content is represented by the sum of the TAG and SE measurements. Integrated band areas were normalized to ergosterol bands. Open/closed symbols: 5/7 μl sample volume. Symbol shapes indicate the biological replicates (B, bottom panel, right). Mean values were compared using an unpaired, two-tailed Welch's *t*-test performed on replicate means. *P*-values <0.05 were considered significant: *P<0.05; **P<0.01; n.s.=not significant. 95% confidence intervals are shown in square brackets.

As a proof of principle, we applied our workflow for comparative analysis of LDs in WT and *sei1*Δ *ldb16*Δ cells. First, we analyzed the cell sizes of these two strains. We found that the *sei1*Δ *ldb16*Δ double-deletion mutant showed a modestly decreased average cell size (∼13% decrease) compared to the WT. This trend and relative correlation of the cell sizes between WT and the double-deletion mutant were confirmed by CASY™ cell volume measurements (∼18% decrease) (Fig. 6A).

Next, we analyzed the mean number of detected LDs in WT and *sei1*Δ *ldb16*Δ cells. As expected from visual inspection of the images, the seipin mutant showed a significantly higher mean number of LDs per cell than the WT (∼41% increase). Furthermore, the image-based analysis also showed that the mean LD area value computed per cell is only slightly increased in the double-deletion mutant compared to the WT (∼11% increase). Since the double-deletion mutant cells are smaller than WT cells, we additionally normalized the LD areas to the cell areas, which accounts for the cell-size differences. When this metric was used, *sei1*Δ *ldb16*Δ cells show an increase of ∼+27% in mean LD area. Semiquantitative data obtained by thin-layer chromatography (TLC), however, showed no difference in NL content (TAG and SE) between the WT and the mutant strain under our cultivation conditions (Fig. 6B; Fig. S8, Table S2).

**Fig. 7.**
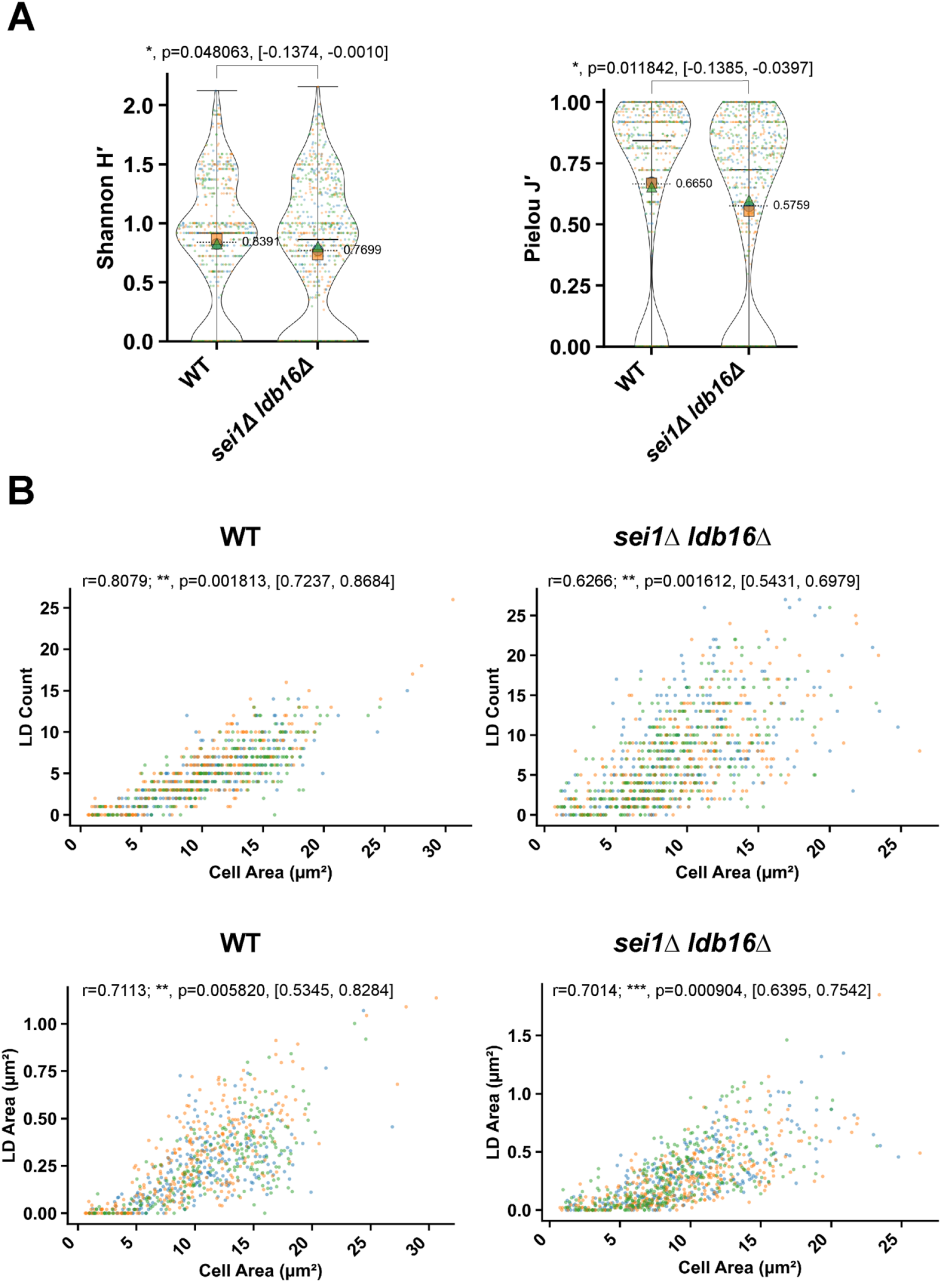
**Image-based quantification of LD-size diversity, evenness and cell-size correlations.** Shannon heterogeneity (Shannon H′) and Pielou's evenness (Pielou J′) plots of LD sizes in WT and *sei1*Δ *ldb16*Δ cells (A). Correlation of LD count and cell area in WT cells (B, top panel, left) and in the seipin mutant (B, top panel, right). Correlation of LD area and cell area in WT cells (B, bottom panel, left) and in the seipin mutant (B, bottom panel, right). Violin plots: solid line, median; dashed line, mean; symbols, replicate means; vertical lines, minimum to maximum; colored circles, individual replicate values. *n*=764 cells; *sei1*Δ *ldb16*Δ, *n*=942 cells. Mean values were compared using an unpaired, two-tailed Welch's *t*-test performed on replicate means. *P*-values<0.05 were considered significant: *P<0.05; **P<0.01; ***P<0.001; 95% confidence intervals are shown in square brackets.

To analyze the overall shape of the LD-size distribution between the two strains, we computed the Shannon entropy (overall diversity of LD sizes) and Pielou's evenness (uniformity of LD area across size classes). Both values were modestly higher in WT than in *sei1*Δ *ldb16*Δ cells, indicating a broader and more balanced distribution of LD-size classes in WT, whereas the double-deletion mutants showed a more concentrated, less diverse LD-size profile (Fig. 7A).

Furthermore, we studied the correlation between the number of LDs and the cell size in WT and the double-deletion mutant. Interestingly, we found that the number of LDs is significantly linked to cell size in WT cells (*r*=∼0.81, *P*=0.001813). In addition, the relatively low variability at each cell-size profile may indicate balanced biogenesis and inheritance of LDs during growth. However, this remains speculative without dynamic or lineage-resolved data. In contrast, this correlation was decreased in the seipin mutant (*r*=∼0.63, *P*=0.001612). The mutant showed greater scatter and heterogeneity at each cell size. Also, the mean area of LDs was linked to the cell size in WT cells (*r*=∼0.71, *P*=0.005820). Again, the spread was relatively consistent across cells. Of note, although double-deletion mutant cells contained significantly more LDs, the correlation of the mean LD area in relation to the cell size was still relatively high in *sei1*Δ *ldb16*Δ cells (*r*=∼0.70, *P*=0.000904) (Fig. 7B).

**Fig. 8.**
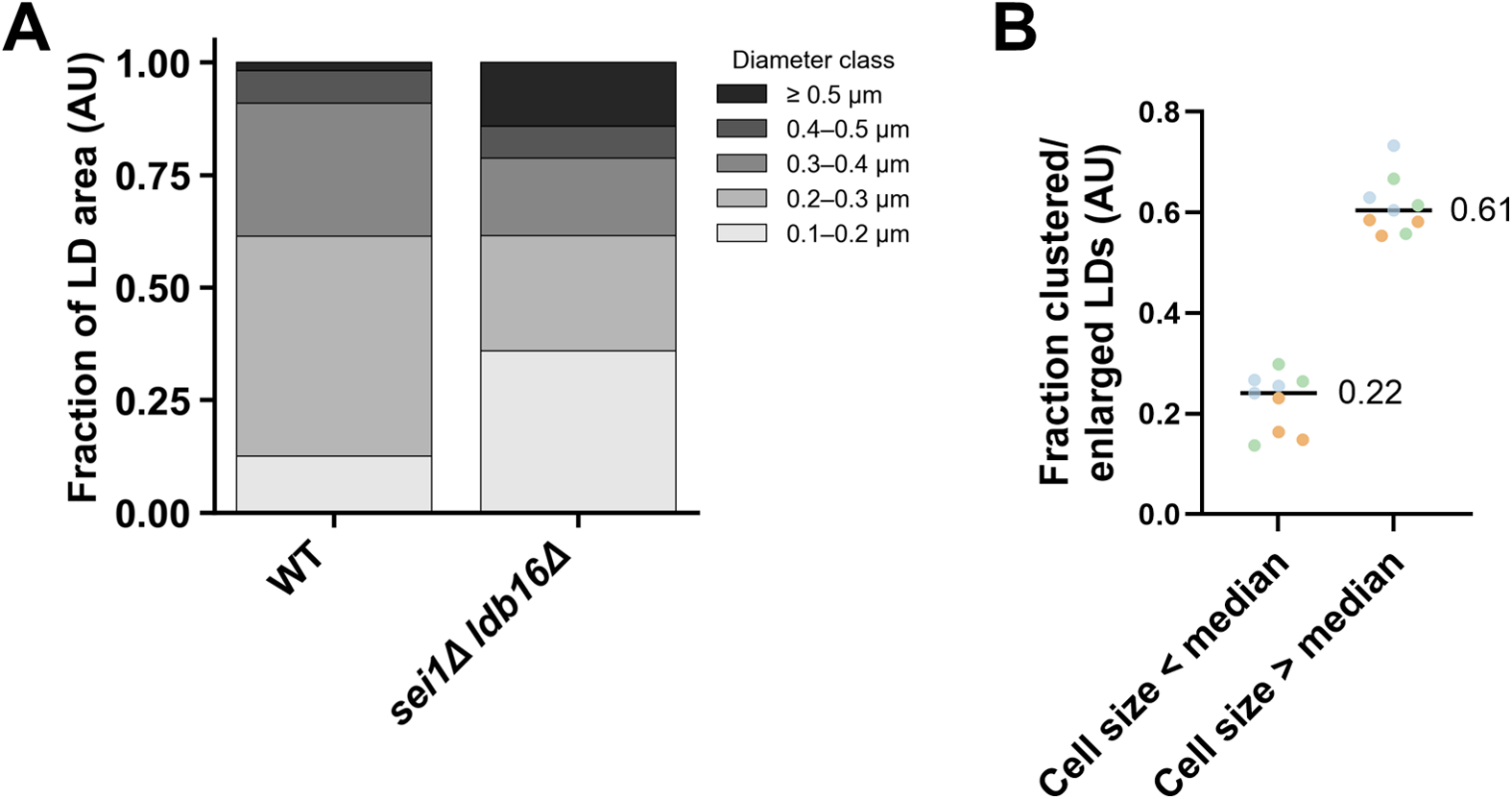
**Distribution of LD-size classes in WT and *sei1*Δ *ldb16*Δ cells.** Stacked plot showing the fraction of the total LD area falling in the defined size classes. A size range of 0.1–0.2 μm is set in the software, but only diameters of ≥0.16 μm are computed (see text). WT, *n*=764 cells; *sei1*Δ *ldb16*Δ, *n*=942 cells (A). Relative abundance of clustered LDs and/or enlarged LDs (>0.4 μm) in smaller and larger *sei1*Δ *ldb16*Δ cells (threshold: median value of the cell area), *n*=942 cells (B).

We next studied the LD-size-class distribution in WT and *sei1*Δ *ldb16*Δ cell populations. In WT cells, the LD area is dominated by LDs with a diameter of 0.2–0.3 μm, which account for ∼49% of the total LD area in the population. About 30% of the LD area is attributable to LDs with 0.3–0.4 μm diameter, ∼13% to LDs with 0.1–0.2 μm diameter, and ∼9% to LDs with ≥0.4 μm diameter (enlarged/supersized). In contrast, *sei1*Δ *ldb16*Δ cells show a striking shift in the occurrence of LD-size classes: ∼36% of the total LD area is attributable to very small LDs (0.1–0.2 μm), the contribution of LDs measuring 0.2–0.3 μm decreases to ∼26%, and the proportion of the area represented by large/supersized LDs (≥0.4 μm) rises from ∼9% in WT to ∼21% in the double mutant (Fig. 8A). Thus, the double deletion does not simply increase LD size uniformly but redistributes NL storage from medium-sized droplets into both very small and supersized LDs consistent with the reduced diversity and evenness of LD sizes indicated by the Shannon size diversity and Pielou's evenness analysis (Fig. 7A). Furthermore, using the data obtained by the SRR method, we quantified the fraction of cells within the population that contained at least one enlarged LD (>0.4 μm in diameter) or an LD cluster (defined as ≥3 LDs with circle edge-to-edge distances of ≤0.2 μm) in *sei1*Δ *ldb16*Δ cells. Furthermore, to account for cell-size differences, we calculated these metrics separately for ‘small’ and ‘large’ cells, using the median cell area of the entire population as the threshold (independent ‘LD_ClusterAnalyser’ module). We found that a large fraction of cells (39% of larger cells and 78% of smaller cells) did not exhibit any of these structures, indicating that the double-deletion mutant shows not only pronounced LD-size heterogeneity at the single-cell level but also substantial cell-to-cell heterogeneity in the occurrence of these phenotypes under our growth conditions (Fig. 8B; Table S3, Fig. S10).

We next applied the histogram-based Otsu and Huang methods to segment LDs from the same datasets, split the segmented particles using a watershed algorithm comparable with the method implemented in Fiji and performed the LD-size and size-class distribution analyses with these alternative methods (independent ‘LD_HistogramSegmenter and LD_HistogramStatistics’ modules). As expected, the LD area per cell and LD-area-to-cell-area values depended strongly on the selected thresholding method. In contrast to results obtained from SRR and chromatographic lipid analysis, the Otsu-segmented data suggested a trend toward reduced LD area per cell (∼−25%) and per cell area (∼−15%) in *sei1*Δ *ldb16*Δ cells compared to WT. Only LDs segmented with the Huang method qualitatively matched the expected overall increase of the LD area in the mutant strain (LD area per cell:∼+8%; LD area per cell area:∼+24%) (Fig. S9A). However, in both cases, the LD area was dominated by the largest LD class in all conditions and failed to reproduce the pronounced redistribution toward very small and supersized droplets in the seipin mutant that was evident with the seeding-based method (Fig. S9B) and upon visual inspection of the deconvolution data.

## DISCUSSION

Our work showed that quantification of subcellular structures, even of simply shaped LDs in yeast cells, is challenging. Our modular workflow aims to systematically mitigate critical sources of bias, such as movement of LDs in intact cells, labeling heterogeneity, potential change in image acquisition settings, or limitations of resolution and contrast enhancement methods.

LD movement is a major source of bias in high-resolution LD-size analysis. Because LDs moved rapidly (Movie 3), fixation was required to immobilize them and avoid repeated imaging of the same organelles during scanning. Faster scanning may partly reduce this limitation, but at the cost of lower signal, especially from small LDs. The short fixation protocol largely preserved LD integrity, including closely associated LDs in the seipin double-deletion mutant. Under starvation, however, WT and mutant cells also contained highly dynamic BODIPY-493/503-labeled vacuolar LDs, consistent with autophagic processes. Although fixation strongly reduced cytosolic LD mobility, these vacuolar structures could not be immobilized at the low fixative concentration needed to avoid LD fusion, probably because low vacuolar pH interferes with crosslinking. Therefore, we included a *z*-stack movement-detection tool that detects and segments most motile LDs for optional interactive exclusion, although some LDs may be missed and occasional noisy structures may be falsely detected.

We used h-maxima to generate initial LD-center seeds and added a 2D PSF-based refinement step to improve center detection, especially for small- and medium-sized LDs. When LD intensities are heterogeneous, or organelles are closely associated or spatially aligned, several maxima can occur very close to each other; therefore, DBSCAN was used to merge dense peaks into an optimized core maximum. Nevertheless, final LD-center detection and registration remain challenging because of intrinsic object properties and postprocessing limitations.

In our workflow, iterative RL deconvolution is used as a preprocessing step to improve image quality, particularly for small and dim LDs (<0.2 μm) that might otherwise escape detection. Image acquisition followed the Nyquist sampling theorem, since maximum optical resolution is essential for imaging small LDs represented by only a few pixels and for deconvolution performance. To maximize temporal resolution, we did not use line averaging, which increased noise; therefore, a mild pre-denoising step with a small kernel was applied to preserve tiny LDs. For deconvolution, we used a measured PSF from 200 nm beads instead of commonly used 100 nm subresolution beads. This bead size matches the dominant LD class in WT cells (∼0.2–0.3 μm), providing a slightly broader and more conservative PSF that still sharpens small LDs while reducing artifacts at small- and medium-sized organelles.

Striking artifacts in enlarged or supersized LDs were removed by machine-learning-assisted adaptive filtering of masked LDs (Fig. 3). The random-forest pixel classifier was effective for this purpose, and we provide a classifier file with several pretrained datasets that can be modified or extended in ilastik. We also included a feature to refine or manually mask artifact-prone regions, which is useful when only a few LDs are affected and batch ilastik segmentation is unnecessary. Since masked LDs are processed with a Gaussian filter, local deconvolution is partly offset in these regions, a bias we consider acceptable. Because RL can alter relative intensities, iterations were limited to 10. This strategy also improved mitochondrial image quality, suggesting broader use under comparable yeast imaging conditions (Fig. S5). Future adaptive deconvolution strategies combining PSF calibration with spatially tuned regularization may further reduce artifacts while preserving very small LDs. The workflow can also be applied to non-deconvolved or alternatively filtered images, making it potentially useful for cell systems with larger LDs, such as mammalian cells.

BODIPY 493/503 also labels PL membranes, including the LD monolayer and other membranes such as the ER (Wolinski and Kohlwein, 2014). Therefore, part of the LD-associated signal comes from membrane-bound dye, which can create a diffuse ‘halo’ and broaden the apparent LD edge compared with reference beads. In addition, 200 nm beads may still appear approximately PSF limited, whereas larger LDs are extended objects with a flat interior intensity profile and cannot be treated as point-like sources. We therefore used a higher radial raytracing threshold for LD detection (50% intensity decay) as a compromise to detect small and larger BODIPY-labeled LDs while limiting size overestimation. Very small objects may also show border intensity cutoffs after deconvolution, limiting their detection. We therefore restricted quantification to LDs measuring ≥160 nm, corrected large LDs (>500 nm) with a fixed factor, applied a ‘flat-stop’ rule for very dim LDs, and included an overlap-based circle cleanup. An interactive viewer allows final refinement of LD registration. Nevertheless, the method cannot recover true LD size but provides an approximation, supported by numerical outputs and registration images for review. Importantly, SRR improved LD detection and separation compared with Otsu and Huang thresholding, which is essential for size-class analysis (Fig. 4; Fig. S9B), and the proof-of-principle results are consistent with published data.

When labeling LDs with BODIPY 493/503, staining consistency may vary across experiments and within individual samples due to LD-size heterogeneity and different cell ages, causing signal-intensity differences. This variability complicates constant photon-detector settings. Consequently, data acquisition often requires photomultiplier tube (PMT) adjustments to keep measurements within the detector's dynamic range. The seeding approach shown here is advantageous because it is independent of absolute grayscale values and histogram profiles, making it robust to intensity variation (Fig. S7). However, photomultiplier adjustments must remain moderate, as strong changes can cause intensity truncation, particularly affecting very small LDs through the intensity offset. Furthermore, BODIPY 493/503 shows affinity for and labels PLs (Wolinski and Kohlwein, 2015). The NL signal is typically much higher than the PL signal, and a low-intensity baseline during detection was sufficient to largely exclude the PL signal from analysis. Furthermore, although BODIPY 493/503 is widely used for NL staining, it may not be effective in detection of nascent LDs; LiveDrop can be used as an alternative fusion marker (Wang et al., 2016). In addition, an increasing number of fluorescent LD probes are available as alternatives to BODIPY 493/503 staining; however, these were not tested in this study (Zhao et al., 2022).

For cell registration, we used the deep-learning ‘YeastSpotter’ method (Lu et al., 2019), which proved sufficient for initial automated detection of yeast cells in sum projections of simultaneously acquired transmission images, whereas, in our setup, this method primarily detects contrast associated with the cell interior, whereas the outer cell boundary is represented less consistently. Installed in a local Python environment, the tool allows efficient batch processing and adjustment of different detection parameters. False-positive detection, such as some intercellular space, or incomplete segmentations can be processed and interactively corrected using a created interactive editor (LD_CellMaskEditor module). Alternatively, images can be processed online with limited batch processing capabilities and user settings at the YeastSpotter web resource. Cell binary images generated by alternative methods, such as Cellpose (Stringer et al., 2021), can also be provided. Registered LDs are automatically assigned to such cell masks, enabling detailed per-cell statistics of LDs within a population of cells, whereas the LD-size classes are defined by the user. Basic statistics include both spreadsheet and graph output including stacked plots to visualize the distribution of LD-size classes in the population (Figs 6–8; Table S1).

Application of our workflow to morphometric analysis of WT and *sei1*Δ *ldb16*Δ double-deletion mutant cells revealed new phenotypic aspects in both strains. Cell size was slightly decreased in *sei1*Δ *ldb16*Δ cells compared with WT, a phenotype not yet described. The mean cell-area ratio from image analysis closely matched cell-size ratios obtained with a cell counter system, supporting the reliability of image-based cell-size determination (Fig. 6A). In yeast, cell size is controlled by mechanisms including cell-cycle regulatory factors (Schmoller et al., 2015), nutrient availability (Talavera et al., 2024), and PL composition (Rao et al., 2018); however, the mechanistic link between reduced cell size and the seipin double-deletion mutant remains elusive.

The mean LD size (area), when normalized to the cell number, is only slightly increased in the *sei1*Δ *ldb16*Δ strain (Fig. 6B) compared to the WT. However, the YeastSpotter cell detection method does not allow us to differentiate between budded and unbudded cells. In this respect, buds were mainly separated and considered as small cells in the segmentation process. Quantification normalized to the cell number does not consider differences in the cell size between WT and mutant cells. Alternatively, the computed LD area was related to the area of the entire cell population. In this regard, the LD area per cell was increased by ∼11% in the mutant strain compared to the WT, whereas the LD area normalized to the cell area was significantly increased in the double-deletion mutant (∼27% increase). Both metrics were higher than the NL data obtained using TLC, where no significant differences between the WT and the mutant were detected. However, estimating yeast NL content from image-based LD-size analysis is limited. MIPs can lose information, especially when spherical LDs overlap axially, and very small nascent fluorescent LDs may not be reliably detected by a given imaging setup. Moreover, NL composition and packing can differ between LDs independently of apparent size. Thus, chromatographic methods such as TLC, although semiquantitative (Knittelfelder and Kohlwein, 2017), remain the standard for more accurate NL determination, whereas LD-size-based imaging provides only a preliminary, coarse estimate.

Interestingly, correlation plots showed that the number of LDs is tightly related to the cell size in WT cells, whereas this correlation is reduced in the seipin mutant. These plots additionally showed that the total LD area is also a function of the cell size in WT. Of note, this correlation is also relatively high for the double-deletion mutant, although the seipin mutant cells show significantly more LDs than WT cells (Fig. 7B). These findings support previous suggestions that seipin is involved in the organization of LD emergence and maintenance (Wang et al., 2016), as well as in the definition of the number of organelles (Choudhary et al., 2020), but only modestly affects overall NL synthesis. Furthermore, a relative increase in LD size with cell size was also detected in an oleaginous yeast using fluorescence-activated cell sorting (Raschke and Knorr, 2009). An open question is how limited size expansion in yeast is controlled. In this respect, we speculate that NL synthesis may be most active at an ER fraction close to the nuclear envelope (Wolinski et al., 2015), such that the timing and duration of the NL synthesis machinery at such a highly active subcellular domain determine how many LDs are generated at seipin-defined ER foci and that this possibly cell-cycle-controlled timing also limits their size expansion. In our model, a fraction of the resulting LDs would then be actively redistributed to other regions of the cells and, in part, inherited by daughter cells as shown previously (Wolinski et al., 2011). The validity of this model will need to be addressed in future studies.

In *sei1*Δ single-deletion mutant, a few supersized LDs in addition to numerous small LDs were observed under synthetic medium conditions (Choudhary et al., 2020; Fei et al., 2008). In addition, a large number of small LDs (<0.2 μm) scattered throughout the cells were observed in transmission electron micrographs of *sei1*Δ cells (Fei et al., 2008). Furthermore, a clear enrichment of small LDs and a lower number of large/supersized LDs were detected in *sei1*Δ *ldb16*Δ cell populations cultivated overnight in SC-ino medium (Renne et al., 2022). We also observed and computed a reciprocal shift in LD size between WT and the *sei1*Δ *ldb16*Δ double-deletion mutant. More detailed, in WT cells, LDs with an estimated diameter of 0.2–0.3 μm dominate and contribute most to the total LD area of the cell population, whereas this class is strongly reduced in the mutant. Conversely, the double-deletion mutant shows a dominant size class of very small LDs (<0.2 μm) and contains enlarged LDs (0.4–>0.5 μm diameter), size classes that are rare in WT cells. The latter LD phenotype of the seipin mutant is likely to be caused by the fusion of LD clusters upon 48 h of prolonged cultivation in rich medium. This uneven size spread is consistent with the Shannon diversity and Pielou's evenness plots (Fig. 7A).

In addition, population-wide analysis of the occurrence of clustered and/or supersized LDs (>0.4 μm) in the seipin mutant revealed that 39% of larger cells and 78% of smaller cells (separated by the median of the cell sizes) do not contain any of these structures (Fig. 8B). This cell-to-cell heterogeneity may be caused by the impaired inheritance of such structures in seipin mutants (Wolinski et al., 2011) and temporally limited cluster formation in the last generation of daughter cells and large, still not separated, buds. However, which underlying mechanisms result in the extreme heterogeneous LD phenotypes in *sei1*Δ *ldb16*Δ cells, i.e. very small organelles scattered throughout the cells and locally enriched LDs, is elusive. Histogram-based segmentation methods are suboptimal for quantitative LD-size analysis. For instance, in a biological sample, overall image intensity can vary significantly even within the same strain, e.g. due to dead or stressed cells, associated heterogeneity in dye uptake, or autofluorescence, but also across experiments. In such cases, the threshold is automatically altered, and the objects are differently registered. In addition, such methods struggle in detecting very small LDs due to threshold limitations, particularly in separating closely associated structures sufficiently. Such factors compromise both overall LD statistics and determination of LD-size-class distribution (Fig. S9). Adaptive/local thresholding can partly compensate for spatial or global intensity differences but remains limited by local intensity statistics and poor separation of closely associated objects (Ljosa and Carpenter, 2009). Histogram- and gray-level thresholding methods are widely implemented in bioimage-analysis workflows, with broader practical limitations discussed in the corresponding literature (Lamprecht et al., 2007; Ljosa and Carpenter, 2009).

Artificial intelligence (AI)-based image quantification has expanded rapidly (Ouyang et al., 2022 preprint), and tools such as StarDist (Schmidt et al., 2018) may be useful for LD detection. However, their outputs depend on model choice, training data, and user settings, while evaluation is constrained by the lack of fully objective ground truth, complicating comparison across imaging conditions (Segebarth et al., 2020). In contrast, SRR uses few principal parameters based on an empirical PSF. Thus, SRR links LD detection more directly to system-specific image formation and may provide a basis for more precise calibrated LD-size quantification. Further refinement would be possible through hybrid synthetic-experimental reference datasets designed to recapitulate both optical image-formation effects, including PSF-dependent blurring and experiment-inherent features. For exploratory comparison, we also implemented support for StarDist Fiji label images in the thresholding module, allowing StarDist-derived LD labels to be analyzed within the same quantitative statistics framework as other segmentation outputs.

Functional morphometric analysis of LD size, number, and spatial distribution is widely used to read out how mutations or treatments reshape lipid metabolism and organelle morphology in yeast and beyond. Our workflow facilitates this analysis and mitigates several key limitations of this process. However, LD quantification remains inherently challenging. In this context, by providing all raw images, intermediate outputs, and analysis scripts as open resource, we see our approach as an experimental platform for community-driven refinement of LD quantification and related image-based analyses.

## MATERIALS AND METHODS

### Yeast cell cultivation

WT BY4741 (*MATa his3*Δ1 *leu*2Δ0 *met*15Δ0 *ura*3Δ0) and Cox4-GFP (*MATa* Cox4-GFP-HIS3MX6 *his3*Δ1 *leu*2Δ0 *met*15Δ0 *ura*3Δ0) were obtained from EUROSCARF, Germany (Winzeler et al., 1999). The *sei1*Δ *ldb16*Δ double-deletion mutant strain (*MATa his3*Δ*1 leu2*Δ*0 met15*Δ*0 ura3*Δ*0 sei1*Δ::*kanMX4 ldb16*Δ::*NatMX*) was constructed in our laboratory (Wolinski et al., 2015). Yeast cells were cultured at 30°C in 5 ml of liquid YPD medium [1% Bacto yeast extract, 2% soya peptone (Roth, Inc., Germany) and 2% dextrose] in six-well microtiter plates in an Eppendorf Thermomixer comfort (Eppendorf, Inc.) at 350 rpm for 48 h. This pre-culture was used for inoculation of 5 ml of fresh YPD to an optical density of 1 (OD_600_) and cultivated for further 48 h at 30°C prior to microscopy.

### Cell-size measurements

Cell sizes of WT and *sei1*Δ *ldb16*Δ cells were determined using a CASY Cell Counter and Analyzer system (OLS OMNI Life Science, Bremen, Germany). For CASY measurements, 5 μl of a 48-h *S. cerevisiae* culture was diluted in 10 ml of CASYton. Four biological replicas were measured, and the mean cell sizes per experiment were determined.

### Cell preparation

Cells were fixed with 1 ml of 2% formaldehyde/1 M sorbitol (pH 7.4) in distilled water for 2 min at room temperature (RT). Subsequently, the cells were pelleted, the supernatant was removed and the pellet was resuspended in 1 ml of 0.5% formaldehyde/1 M sorbitol containing 5.1 μM BODIPY 493/503 (Invitrogen, Inc.). After 20 min of staining at RT, cells were mounted without additional washing on standard microscope slides and covered with a 0.17 mm±5 μm coverslip (Marienfeld, Inc., Germany).

### High-resolution 3D image acquisition

Imaging was performed using a Leica SP8 DLS confocal microscope with spectral detection (Leica Microsystems, Inc.) and a Leica HCX PL APO 63× oil-immersion objective with a numerical aperture of 1.4. Images were acquired at a scan speed of 600 Hz in unidirectional mode. We computed an optimized sampling density (Pawley, 2006) according to the Nyquist sampling theorem using the open-source sampling calculator provided by Scientific Volume Imaging (SVI, Inc.; https://svi.nl/Nyquist-Calculator) of 40×40×120 nm (x/y/z) (41×41 μm image size at 1024×1024 scan format). BODIPY 493/503 was excited at 488 nm, and emission was detected between 500 and 550 nm using a standard PMT. 8-bit fluorescence and transmission images were acquired simultaneously.

### Generation of an empirical PSF

For generation of a 3D PSF for RL deconvolution, we distilled the PSF from spatially acquired 200 nm fluorescent beads [same settings as for LD imaging, 40×40×120 nm (x/y/z) sampling] using a custom Python tool. In brief, the bead extraction process includes the application of the Otsu method to segment the beads in the 3D stack, computation of the center of mass of detected objects, and application of a Gaussian filter for smoothing (σ=0.7), thus slightly regularizing the original PSF, merging the beads, centering the peak voxel but not the mass (i.e. preserving the bulk intensity distribution) and normalizing the extracted PSF. In total, eight beads were merged for PSF generation.

### Deconvolution

Deconvolution was performed using the RL algorithm (Richardson, 1972; Lucy, 1974) with entropy-based regularization (Sibarita, 2005), computational acceleration and additional optional graphic processor unit (GPU) acceleration. The raw datasets were pre-filtered using 3D Gaussian filtering (σ=1) and post-filtered using σ=0.5. RL deconvolution was performed using a low number of ten iterations and the distilled empirical 3D PSF.

### Machine-learning-supported deconvolution artifact batch segmentation

For machine learning and masking of large LDs subjected to deconvolution artifacts, ilastik and the parallel random-forest (scikit, scikit-bio development team, 2025; https://scikit.bio/) classifier were used (Berg et al., 2019). Installation of ilastik was performed according to the developer's instructions (https://www.ilastik.org/documentation/). The following parameters for classification of imported deconvolution data were selected: color/intensity – Gaussian smoothing; edge – Laplacian of Gaussian, Gaussian gradient magnitude, difference of Gaussian; and texture – Hessian of Gaussian eigenvalues. All features were applied with σ=1.60^−10^. LDs were trained (autocontext two-stage) by marking LDs in the 2D projections showing deconvolution artifacts using the brush tool. MIPs of deconvolution data were transferred to the batch processing window in ilastik and computed. The segmentation results (masks; simple segmentation stage 2) were exported in 8-bit *.tif format for further use in the Python workflow. The following basic binary operations were performed to refine the 3D segmentation results using the LD_RingingCleaner module: Fill holes, Close, Remove outlier (radius=2, threshold=20), and Gaussian filter (σ=1; adaptive). Adaptive filtering was performed using the LD_AdaptiveFiltering module, with Gaussian filtering (σ=4) applied to masked LD areas, transition (edge) Gaussian filtering (σ=2), and no filtering for exterior regions.

### LD-center detection and LD registration

Local intensity maxima were detected using the h-maxima method (van der Walt et al., 2014) with subpixel accuracy. The DBSCAN algorithm (Ester et al., 1996) was applied to merge densely associated maxima and, in this way, to refine the maxima positions. For further correction of LD centers, template matching was performed by comparing a small region around the seed with the MIP of the empirical PSF template. The template was shifted in subpixel steps within the region of interest. The shift that gave the best match between image and PSF computed using invariant least-squares template matching was taken as the ‘correct’ center. The criteria for LD detection by radial raytracing are described in the text and in Fig. S2.

### Cell registration

For registration of yeast cells in transmission images, YeastSpotter, a deep-learning approach, was used (Lu et al., 2019). In brief, the YeastSpotter library was installed in our local Python environment (https://github.com/alexxijielu/yeast_segmentation). Sum projections of acquired 3D transmission data, simultaneously acquired with the fluorescence data, were created and batch processed by running a custom script in Jupyter Notebook 7.2.2 (https://docs.jupyter.org/). Afterward, the resulting segmentation results were refined using the LD_CellMaskRefiner and LD_CellMaskEditor modules.

### PC hardware

Computations were performed using an Intel Core i9-14900KF, 8xP, 16xE, 36MB L3 Cache CPU, 96GB DDR5-6000 RAM and an NVIDIA GeForce RTX 4090 graphic card with Compute Unified Device Architecture (CUDA) for GPU acceleration.

### Python platform

The coding of Python scripts was performed using the Scientific Python Development Environment (Spyder) IDE (spyder-ide.org; Python 3.12.3 64-bit, Qt 5.15.2, PyQt5 5.15.10, Windows 11). Spyder was installed and launched via Anaconda Navigator 2.6.3 (https://www.anaconda.com/). Coding was assisted by Chat-Generative Pre-Trained Transformer (ChatGPT; OpenAI.com).

### Lipid extraction and NL analysis

Lipids were extracted as described previously (Matyash et al., 2008). Nonpolar lipids were separated by TLC using a solvent system of petroleum ether/diethyl ether/acetic acid (70:30:2, v/v/v). NL bands on TLC plates were quantified by densitometry based on peak area using a CAMAG TLC scanner at 546 nm, with triolein (Nu-Chek Prep, Elysian, MN) as the standard for TAGs, cholesteryl oleate for SEs, and ergosterol as an internal reference. NL amounts were normalized to ergosterol content.

### Statistics

Statistical analyses were performed using GraphPad Prism 10 software (Dotmatics, Inc.) or using the created ‘LD_Statistics’ Python module (LD Toolbox) and the LD_SegementerStatistics module (histogram-based segmentations) as indicated in the text. Statistical significance between strains was assessed using a two-sided unpaired Welch's *t*-test performed on replicate means with confidence levels indicated by asterisks. *P*-values <0.05 were considered significant: *P<0.05; **P<0.01; ***P<0.001. Three image stacks per sample were acquired for analysis. Experiments were performed in three biological replicates unless otherwise stated in the text. The Shannon diversity and Pielou evenness values were computed in Python using the scikit-bio package (https://scikit.bio/).

## Supplementary Material



10.1242/biolopen.062429_sup1 Supplementary information

Table S2. TLC scanner densitometry values and statistics.

Table S3. Automated cluster and enlarged/supersized lipid droplet analysis values.
