# Supplementary material for: Lipid droplet size profiling in yeast
Source: Biol Open. 2026 Jul 9;15(7):bio062429. doi: 10.1242/bio.062429 (PMC13383019; doi:10.1242/bio.062429)
Supplement: Supplementary information [file biolopen-15-062429-s1.pdf]

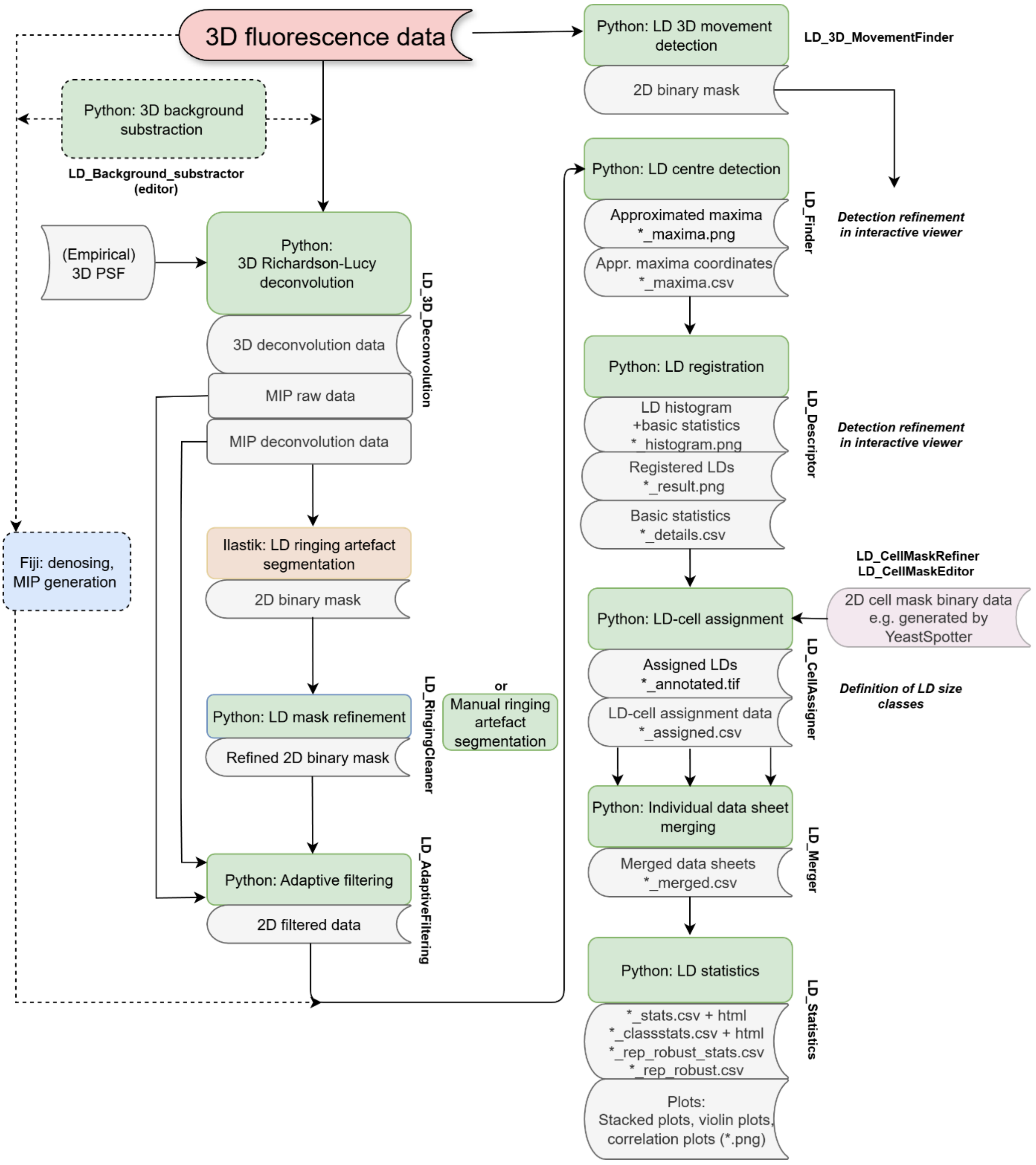

Fig. S1. Workflow for quantifying lipid droplets in yeast cell populations. The process involves steps using Python scripts (green fields), the open-source software Ilastik (orange field), Fiji (alternative process; blue field), and the deep learning tool "YeastSpotter" for cell registration (light magenta field). Generated output data of the individual modules are shown in the gray fields. The 3D deconvolution and adaptive-filtering part can be bypassed on-demand (dotted lines).

Biology Open • Supplementary information

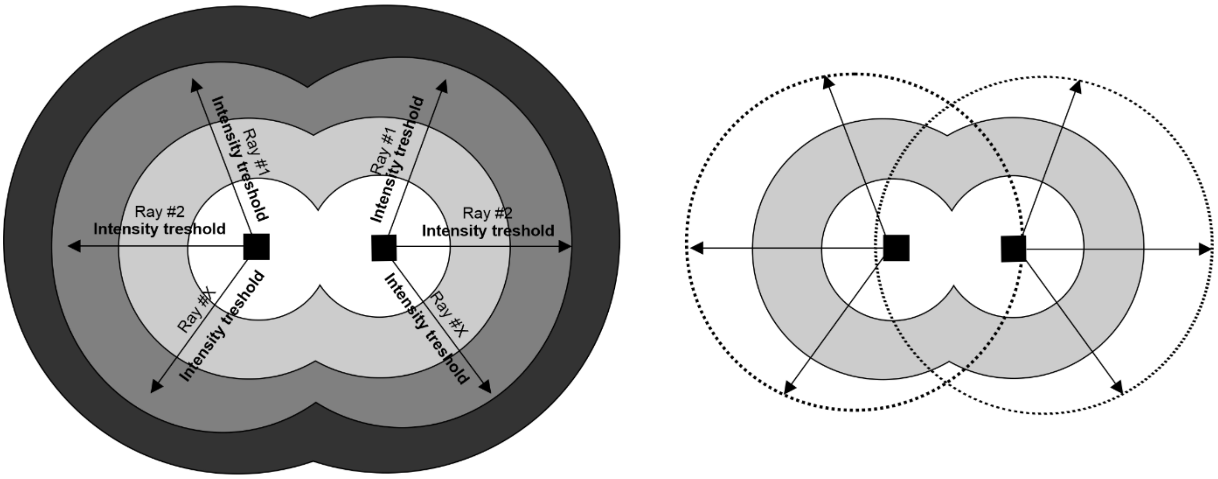

Fig. S2. Basic principle of seeding-based radial raytracing lipid droplet detection. Initially, object centers are approximated and used as seeds. A defined intensity threshold along a user-defined minimum number of virtual rays from the object's intensity maximum is set as criterion to stop the registration process (left image). Using these stopping criteria, circles are computed to define the LDs (right image, dotted lines). This principle enables the detection of closely associated objects, where the intensity drop between object intensity maxima is low. The intensity maxima are computed by the "LD_Finder" module; the radial raytracing procedure and LD registration are conducted using the "LD_Descriptor" module.

Biology Open • Supplementary information

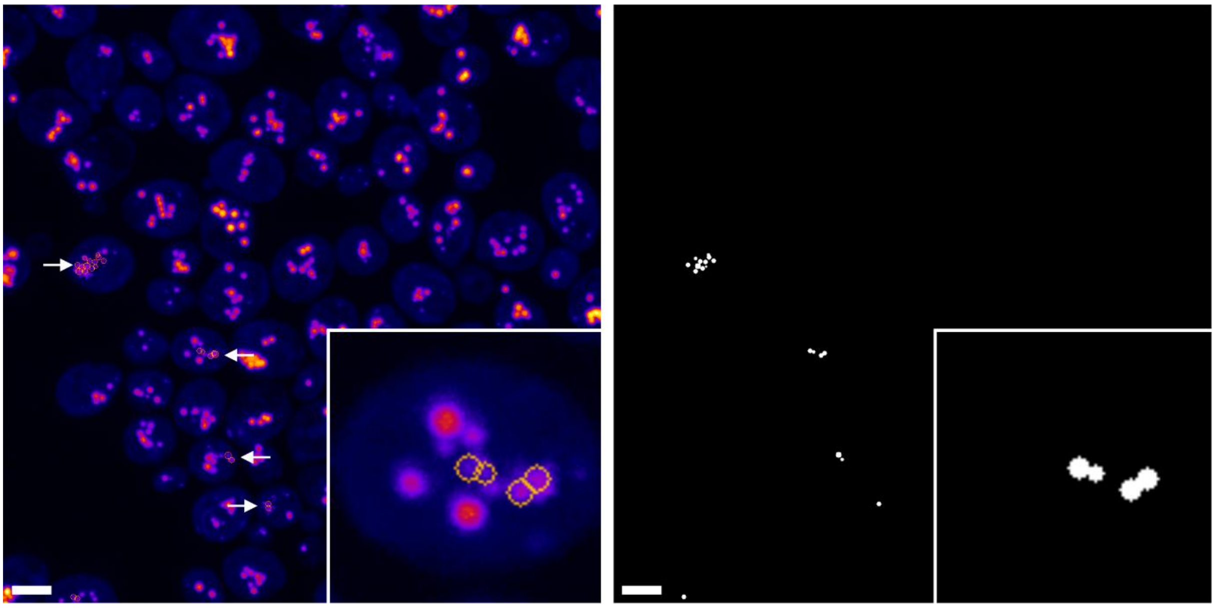

Fig. S3. Detection of residual lipid droplet movement in z-stacks of fluorescently labeled fixed wildtype cells. Moving LDs are indicated by orange circles (arrows, left image). Segmented moving LDs are used for further refinement of the LD detection process (right image). Insets represent enlarged regions of the images. Bar = 5 µm.

Biology Open • Supplementary information

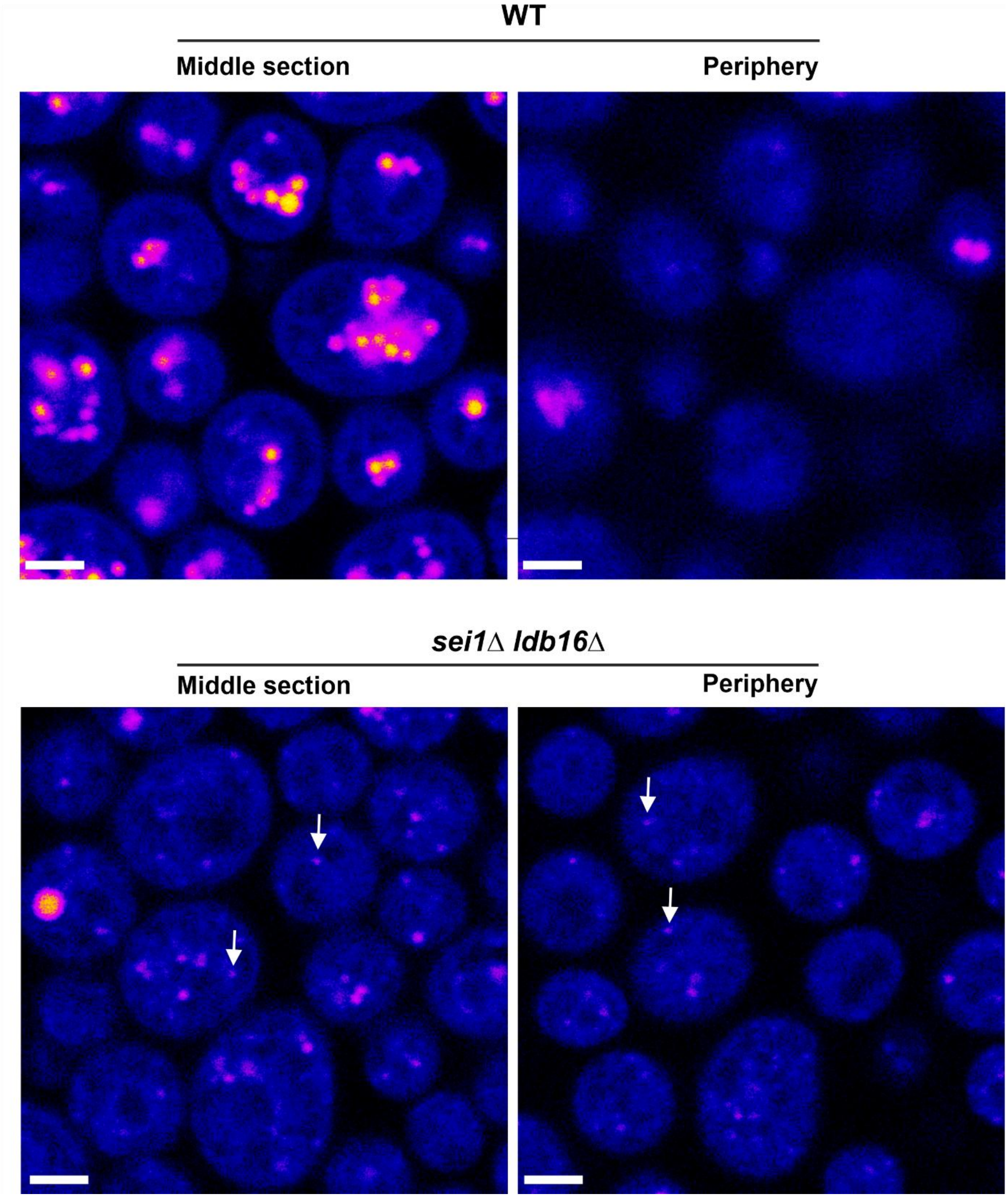

Fig. S4. Lipid droplet phenotypes in wildtype and *sei1*Δ *ldb16*Δ cells. Occurrence of tiny, spatially scattered LDs in *sei1*Δ *ldb16*Δ cells (bottom panel, arrows) which are mainly absent in wildtype cells (top panel). Single optical sections from the middle and the periphery of the cells. Brightness of the images was increased for display. Bar = 5 µm.

Biology Open • Supplementary information

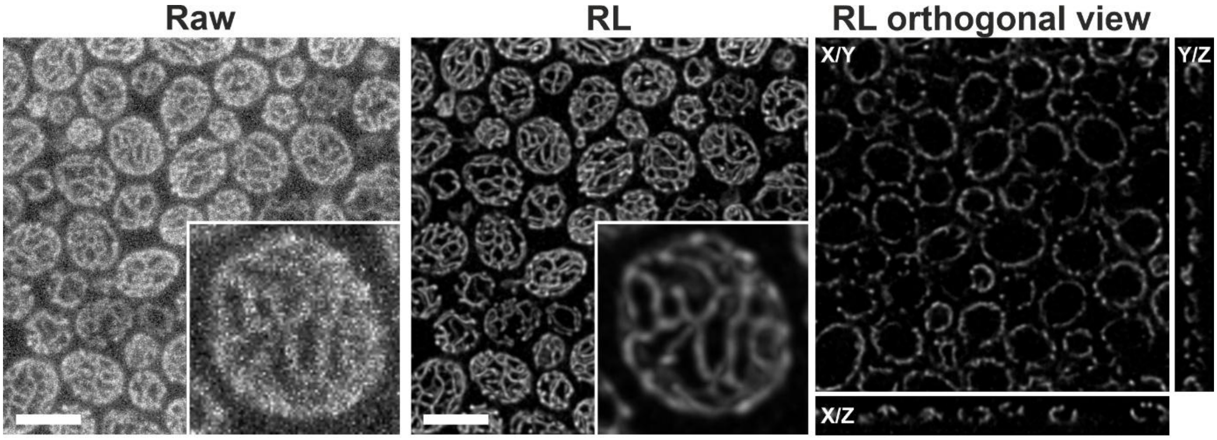

Fig. S5. Improvement of resolution, contrast, and overall image quality with Richardson-Lucy deconvolution and a measured point-spread-function. MIP of 3D raw data showing yeast cells endogenously expressing Cox4-GFP (mitochondrial marker) (left image). MIP of the deconvolution data, 10 iterations; middle image. Orthogonal views of the deconvolution data showing the improved axial resolution of the dataset (right image). Raw data was acquired using 600 Hz and without line averaging. Bar = 5 µm.

Biology Open • Supplementary information

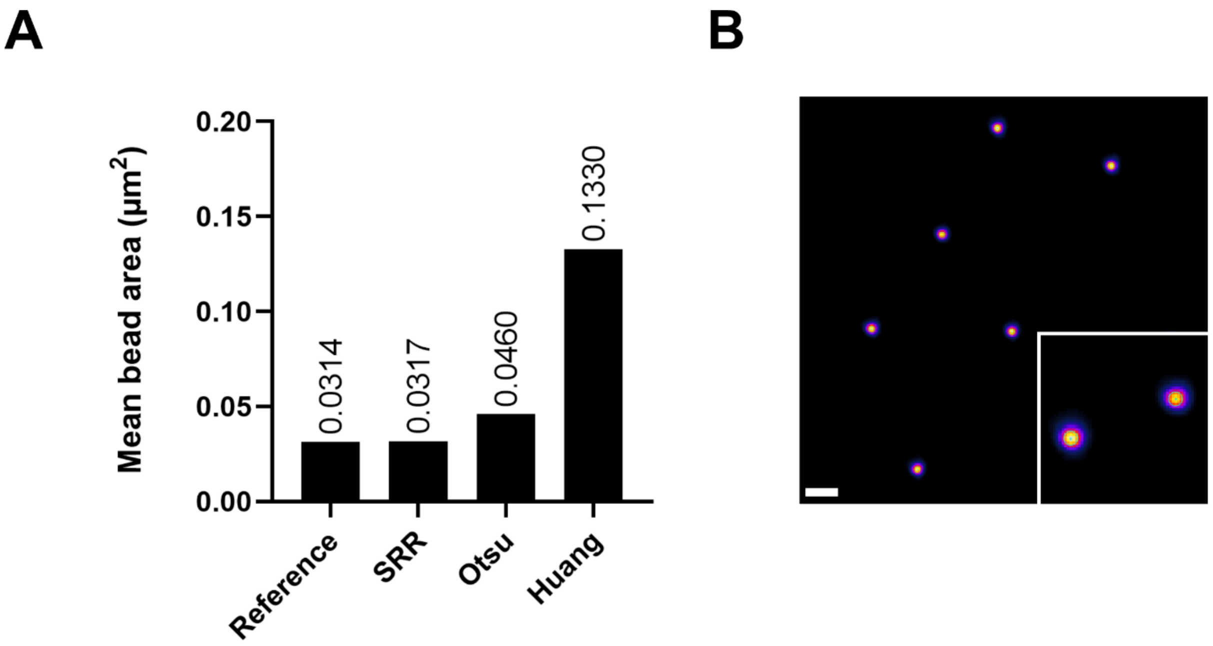

Fig. S6. Quantification of deconvolved 200 nm fluorescent beads using seeding-based radial raytracing as well as using Otsu and Huang segmentation. Computation of the mean bead area using the different methods. The calculated reference size of the beads with a 0.2 µm diameter was determined using the formula: $A = \pi(d/2)^2$. Absolute values are displayed at the top of the bars in graph **(A)**. MIP of eight 200 nm fluorescence beads used for quantification. Inset represents an enlarged area of this image **(B)**. Bar = 1 µm.

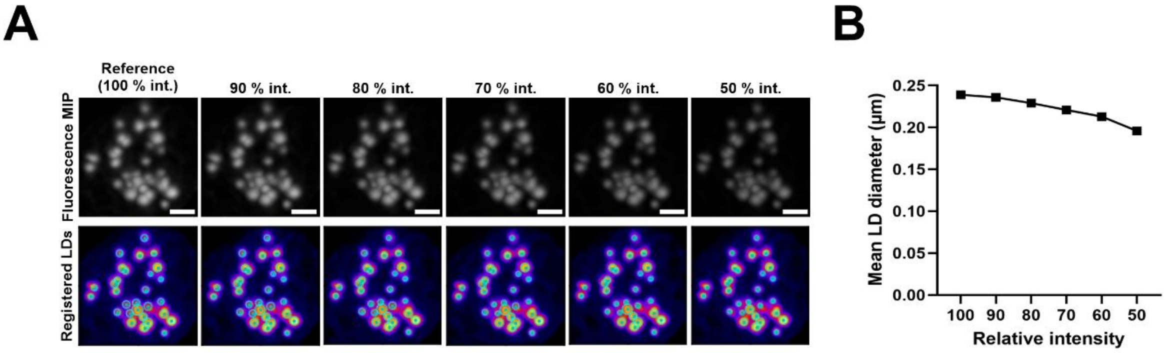

Fig. S7. Performance of the seeding-based radial ray tracing lipid droplet registration approach with altered object intensity. Image series of LDs in a single WT cell with progressively reduced gray level intensities relative to the reference (100 % intensity), (top panel). Registered LDs identified using the seeding-based registration approach. Images are intensity scaled only for display, (bottom panel) **(A).** Graph depicting the computed decrease in mean LD diameters as image intensities are reduced **(B)**. Bar = 1 µm.

Biology Open • Supplementary information

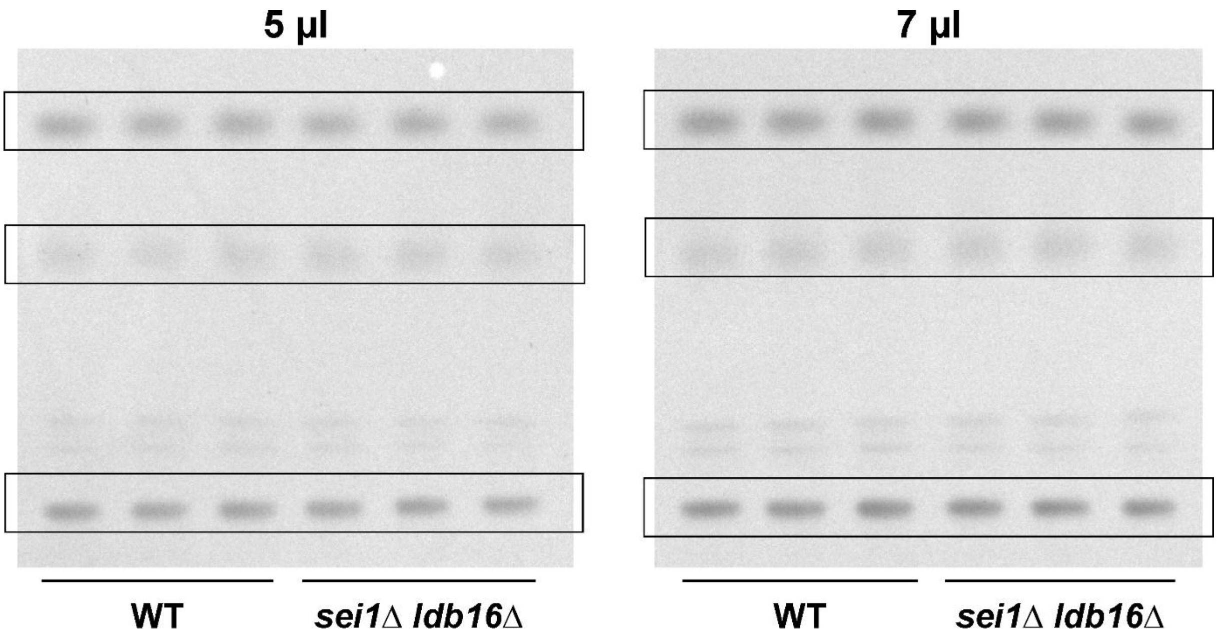

Fig. S8. Thin-layer chromatography of lipid extracts from wildtype and *sei1*Δ *ldb16*Δ cells. TAG and SE band intensities were quantified by densitometry and normalized to the corresponding ERG band. Bands from 5 µl extract per lane (left image) and 7 µl extract per lane (right image). Experiments were performed in three biological replicates for each strain. Experiments were performed in three biological replicates for each strain.

Biology Open • Supplementary information

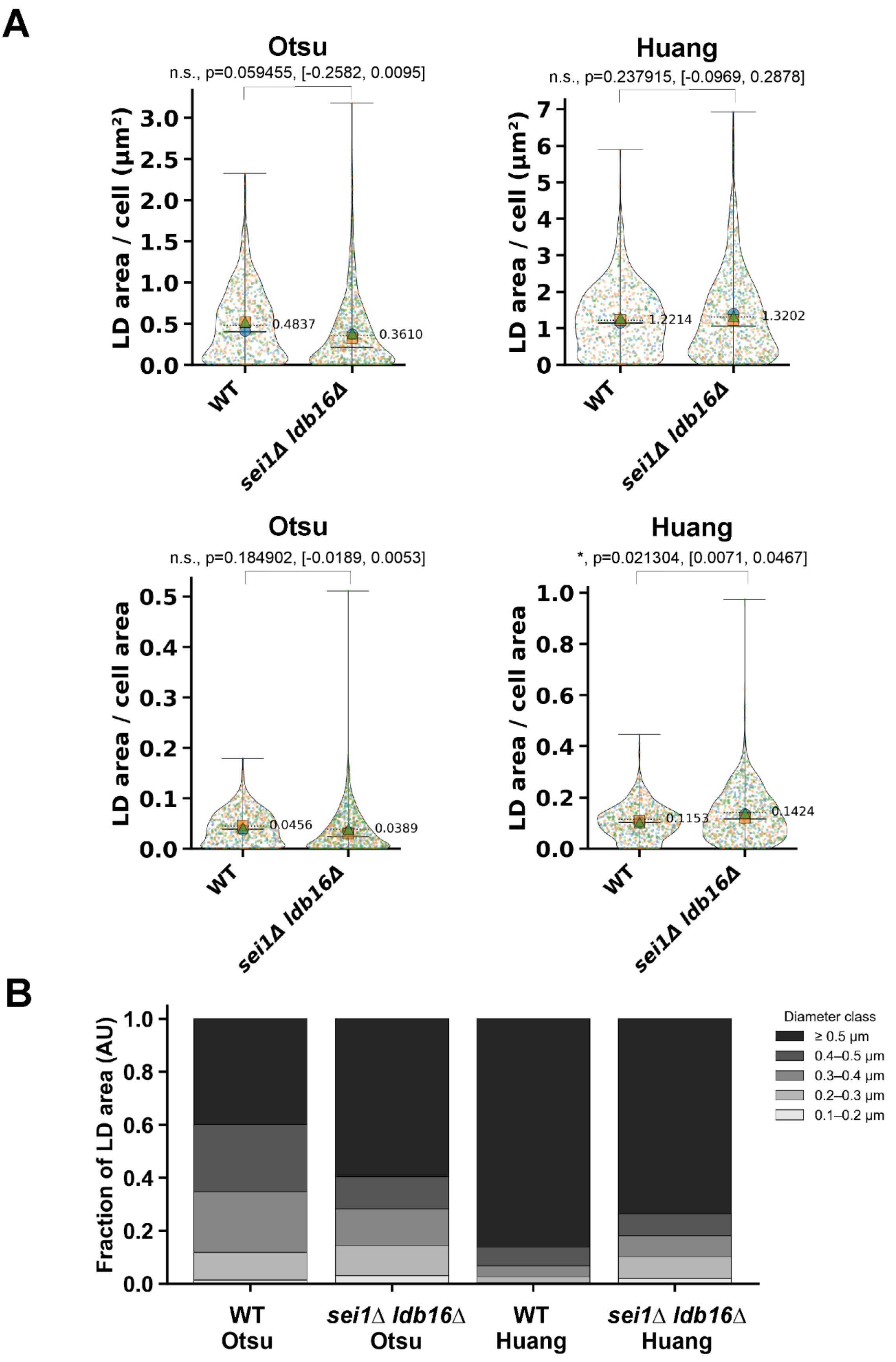

Biology Open • Supplementary information

Fig. S9. Morphometric analysis of lipid droplets using histogram-based Otsu and Huang segmentation. Plots of LD sizes in WT and *sei1*Δ *ldb16*Δ cells (LD area normalized to either the cell number or the total cell area) obtained using Otsu and Huang segmentation. Violin plots: solid line, median; dashed line, mean; symbols, replicate means; vertical lines, min–max; colored circles, individual replicate values. Mean values were compared using an unpaired, two-tailed Welch's t-test performed on replicate means. P-values < 0.05 were considered significant: * $p < 0.05$; ** $p < 0.01$; *** $p < 0.001$; 95% confidence intervals are shown in brackets. **(A)**. Plotted LD area fractions of the populations obtained using either Otsu or Huang segmentation in the two strains and using the defined LD size classes indicated in the legend. Size class (diameter) values are based on computing the equivalent circular diameters from the segmented 2D particles area (formular: $d=2\text{sqrt}(A/\pi)$) **(B).**

Biology Open • Supplementary information

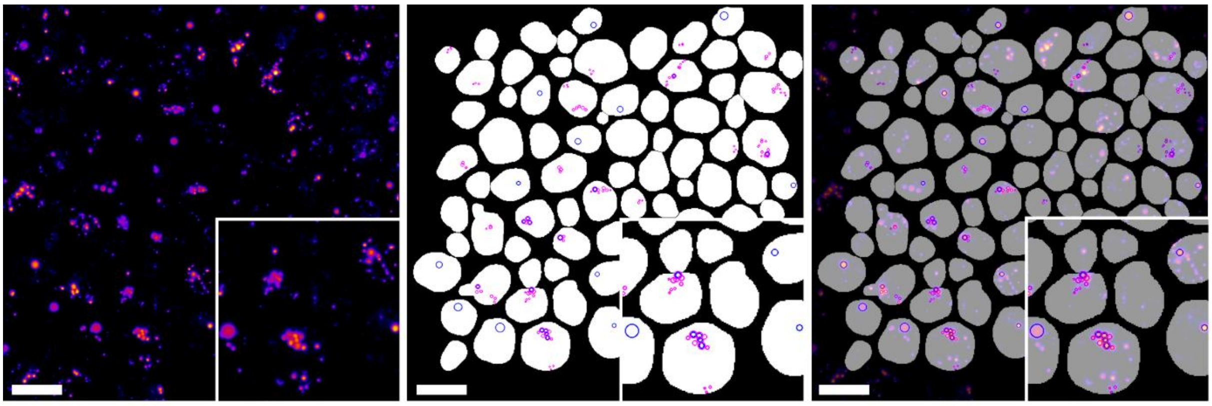

Fig. S10. Detection of lipid droplet clusters and enlarged LDs in *sei1*Δ *ldb16*Δ cells. MIP of adaptively filtered data (left image). Detected clusters are indicated by magenta circles as well as enlarged LDs (>0.4 µm) by blue circles (middle image). Overlay of the two data sets (right image). Bar = 5 µm.

Table S1. Description of Python scripts (modules).

Module	Functions	Input	Output
LD_WorkflowManager	Graphical user interface to define a root folder, create a predefined folder tree, and start the different modules.	-	config.json (root-definition config file)
LD_MovementFinder	Detects LD movement in z-stacks (optionally GPU-accelerated). The segmented 2D data can be used in the editor of the LD Finder module to refine LD center detection.	-Raw fluorescence z-stack	-Overlay z-stack, including labels to highlight moving LDs.<br>-MIP of the overlay z-stack.<br>-MIP of segmented LDs.
LD_3D_Deconvolution	Performs 3D Richardson-Lucy deconvolution on imported z-stacks optionally GPU accelerated). Requires a measured PSF (provided).	-Raw fluorescence z-stack<br>-Measured PSF	-Deconvolved z-stack.<br>-MIP of deconvolved z-stack.<br>-MIP of raw data.
LD_RingingCleaner	Converts and refines binary masks of LDs showing ringing artefacts derived from Ilastik or other software. Includes a manual ringing-artefact masking editor.	- (Ilastik) binary masks	-Refined 2D binary masks.
LD_AdaptiveFiltering	Compensates for deconvolution-induced ringing artefacts.	-Refined 2D binary masks<br>-MIP of raw data<br>-MIP of deconvolved z-stacks	-2D adaptively filtered data.
LD_Finder	Detects centers of LDs in fluorescence images. Includes an interactive editor for detection refinement.	-(Adaptively) filtered 2D data	-2D maxima image<br>-Spreadsheet including LD center coordinates
LD_Descriptor	Describes LDs by circles based on the seeding-based radial raytracing approach. Includes an interactive editor for LD registration refinement. Numerical output enables morphometric analysis of LDs.	-MIP of deconvolved z-stacks<br>-Spreadsheet showing object center coordinates	-Overlay 2D image of registered LDs.<br>-Histogram of LD diameter.<br>-Spreadsheet including LD number, diameter and area.
LD_TransmissionProjector	Creates filtered sum projections from 3D transmission data required for the cell registration process.	-Raw transmission z-stacks	-Sum projections of transmission z-stacks.

Biology Open • Supplementary information

LD_CellMaskRefiner	Automatically refines binary cell masks created by "Yeast Spotter" or other software.	-2D cell masks	-Binary cell masks (cell areas).
LD_CellMaskEditor	Interactive editor to refine cell masks manually.	-2D cell masks	-Binary cell masks (cell areas).
LD_CellAssigner	Assigns registered LDs to individual cells. Allows the definition of LD size classes.	-2D binary cell masks<br>-Spreadsheet created by LD_Descriptor	-Spreadsheet including per-cell assigned object values<br>-Overlay images showing LDs assigned to individual cells, color-coded by LD class.<br>-size_classes.json (size class config file)
LD_FileMerger	Merges spreadsheets of individual z-stacks, generated by LD_CellAssigner, into a single file.	-(Multiple)spreadsheets.	-Merged spreadsheet(s).
LD_Statistics	Generates LD statistics including basic plots.	-Merged spreadsheet(s) of a single or multiple samples<br>-size_classes.jason (config file created by LD_CellAssigner.	-Spreadsheets including descriptive statistics, per-size class statistics, significance (in case of comparative statistics; Unpaired Welch's t-test and others, Violin plots (LD number/cell; LD number/cell area; LD area/cell; LD area/cell area; Shannon (heterogeneity) H'; Pielou (evenness) J'.<br>-Correlation plots (LD number vs cell areas; LD area vs cell area).<br>- *. mpflfig Matplotlib file. Enables subsequent editing of the plots (e.g. style) using Python.<br>Spreadsheets detail:<br>_stats.csv/html (global statistics),<br>_classstats.csv/html (size-class statistics),<br>_replicate_stats.csv/html (replicate-level statistics),<br>_rep_robust.csv/html (replicate-robust statistics),

Biology Open • Supplementary information

			violin_symbol_color_key.csv.
LD_ClusterAnalyser	Detects LD cluster and supersized LDs. Not included in the workflow manager. Includes a help file.	*_details.csv files generated by the LD_descriptor_v1 module.<br>* (refined) cell mask images.	-Spreadsheets with LD values assigned to cells.<br>-Overlay images showing the detected LD clusters and/or supersized LDs.<br>- Summery spreadsheet.
LD_Segmenter	Performs “standard” histogram-based Otsu and Huang segmentation or manual thresholding as well as watershed object separation in batch mode. Alternatively, can import and use 16-bit StarDist labels generated in Fiji. Not included in the workflow manager. Includes a help file.	-Folder containing MIPs of fluorescentely labeled LDs.<br>-Cell mask folder.	-Spreadsheets containing cummulative and detailed values: size_classes.json (size-class settings), _results.csv (single-LD particle results), _summary.csv (per-cell summary), LDseg_noWS.png (LD segmentation before watershed), _LDseg.png (StarDist LD label preview), _cells_LDs_overlay.png (cell/LD overlay with boundaries and LD centroids), _input.csv (input file for the LD_SegmenterStatistics module).
LD_SegmenterStatistics	Performs statistical analysis (plots, spreadsheets) using the LD_Segmenter output. Include a grouping feature for superplot generation. Not included in the workflow manager. Includes a help file.	-*_input spreadsheet(s).	See LD_Statistics output.

Table S2. TLC scanner densitometry values and statistics.

Available for download at
https://journals.biologists.com/bio/article-lookup/doi/10.1242/bio.062429#supplementary-data

Table S3. Automated cluster and enlarged/supersized lipid droplet analysis values.

Available for download at
https://journals.biologists.com/bio/article-lookup/doi/10.1242/bio.062429#supplementary-data

Biology Open • Supplementary information

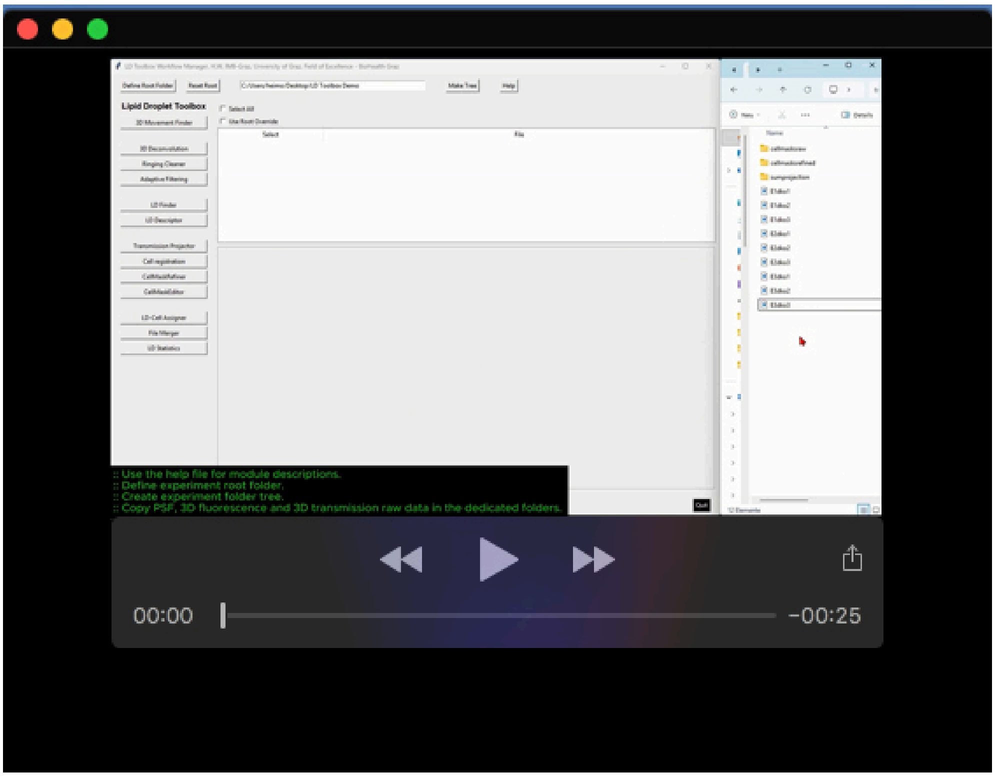

Movie 1. LD quantification workflow.

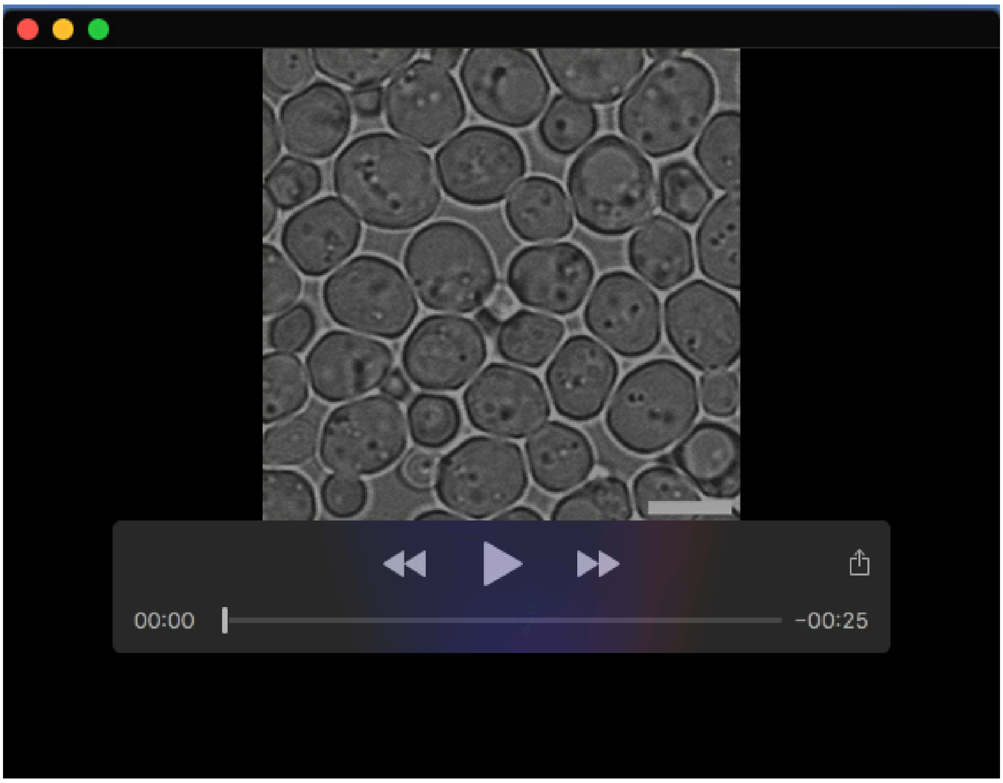

Movie 2. LD motility in wild-type cells.

Biology Open • Supplementary information

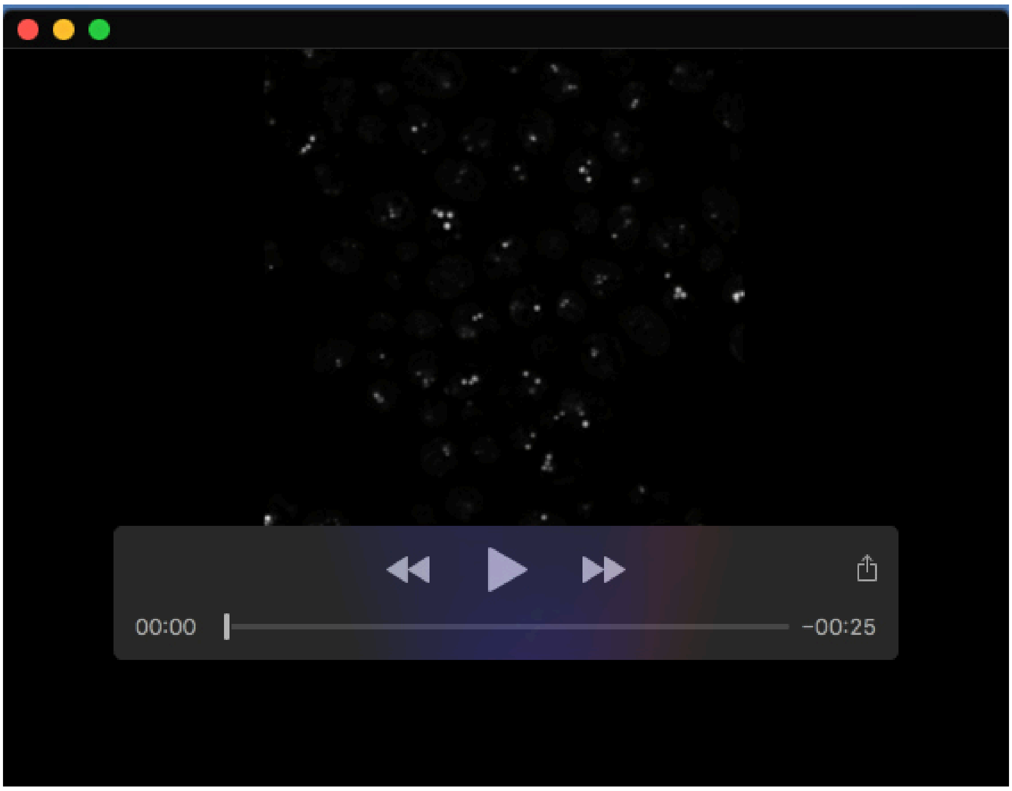

Movie 3. Detection of LD movement in 3D stacks.

Biology Open • Supplementary information
