# Supplementary material for: Lipid droplet size profiling in yeast
Source: Biol Open. 2026 Jul 9;15(7):bio062429. doi: 10.1242/bio.062429 (PMC13383019; doi:10.1242/bio.062429)
Supplement: Supplementary file 1 [file biolopen-15-062429_review_history.pdf]

Lipid droplet size profiling in yeast

Katharina Gritsch, Klara Skrobar Martincic, Kristian Bredies and Heimo Wolinski
10.1242/bio.062429

Editor: Catherine L. Jackson

Review timeline

Original submission:	30 January 2026
Editorial decision:	6 February 2026
First revision received:	7 May 2026
Editorial decision:	1 June 2026
Second revision received:	3 June 2026
Accepted:	9 June 2026

Original submission

First decision letter

MS ID#: bio.062429

MS Title: Lipid droplet size profiling in yeast

Authors: Katharina Gritsch; Klara Skrobar Martincic; Kristian Bredies; Heimo Wolinski

I have now reached a decision on the above manuscript.

The reviewer reports are shown at the bottom of this email.

As you will see, the reviewers were positive but raised a few criticisms that prevent me from accepting the paper at this stage.

They suggest, however, that a revised version might prove acceptable, if you can address their concerns. If you think that you can deal satisfactorily with the criticisms on revision, I would be pleased to see a revised manuscript. We would then return it to the reviewers.

At this stage, we also ask you to ensure your manuscript complies with our formatting guidelines. Provided you are able to fully address the referees' comments, we are positive about publication of your paper (we accept over 95% of revision submissions) and therefore hope you won't mind any extra work involved in reformatting your manuscript at this point.

Please upload both a 'clean' version of your Word file, along with a highlighted version clearly showing where you have made changes in the revised manuscript. Please avoid using 'Track changes' in Word files as these are lost in PDF conversion.

I should be grateful if you would also provide a point-by-point response detailing how you have dealt with the points raised by the reviewers in the 'Response to Reviewers' box. Please attend to all of the reviewers' comments. If you do not agree with any of their criticisms or suggestions please explain clearly why this is so.

Reviewer 1

© 2026. Published by The Company of Biologists under the terms of the Creative Commons Attribution License (https://creativecommons.org/licenses/by/4.0/).

Comments for the author

Gritsch et al describe a method that employs the use of radial raytracing and supervised machine learning to explore lipid droplets in yeast. Specifically, comparing wild type and mutant cells lacking the Sei1-Ldb16 seipin complex. The study is well thought out and easy to follow, with a clear and concise conclusions. This study will be a valuable contribution to the field, and I agree with the conclusion of the paper that "we see our approach as an experimental platform for community-driven refinement of LD quantification and related image-based analyses". While overall the manuscript is well written I have noted a few grammatical inaccuracies and areas that could improve impact of the manuscript.

Materials and Methods

1. Line 252 remove less than or equal to sign and replace with less than sign.

Figures

1. To improve visualisation and interpretation, add trend lines to individual graphs of figure 7B and p-values for correlation analysis. Further to this, p-values should be added in the text next to the values they relate to, for example, on line 417 a r-value of -0.81 is in the brackets only.

Discussion

1. In the literature do you see in sei1Δ ldb16Δ double deletion mutant cells a similar magnitude of effect when compared to your model partnered with your analysis technique? Are there comparable LD analyses that you could cite as a direct comparator?

2. Line 558 authors suggest an alternative to BODIPY 493/503 staining. I wonder if whether it's worth mentioning that fluorescently labelled fusion protein that are LD specific could also be used as an alternative especially to explore nascent LDs that may be poorly detected with BODIPY 493/503.

A few minor suggestions to improve readability

1. Line 554 remove "also"

2. Line 608 minor grammatical error I believe it should read "be most active at ER fractions close to the nuclear envelope..."

3. Line 635 I would remove the words "poorly useful" and replace with suboptimal

<u>Reviewer 2</u>

Comments for the author

1. Experimental quality

a. Does each figure have the proper controls?

* In Figure 5 the authors assign LD to yeast cells, but they don't show LD: the reader cannot visually verify whether the assignment of LDs to segmented cells is accurate. Overlaying the LD channel with the transmission image, or providing zoomed-in insets showing LD localization, would greatly improve the interpretability of this figure.

b. Are experiments performed using appropriate methods that will answer the question (or test the hypothesis or support the observations) posed by the authors? Is the right tool used for the job?

* Overall, the imaging and analysis approaches are appropriate for the authors' stated goals.

© 2026. Published by The Company of Biologists under the terms of the Creative Commons Attribution License (https://creativecommons.org/licenses/by/4.0/).

* In Figure 7A the use of Shannon and Pielou indices is unconventional for organelle distribution analysis (and strongly dependent on binning choices). While creative, the authors should justify why these ecological metrics are superior to standard distribution descriptors (e.g., CV or Skewness) for LD populations. To describe LD heterogeneity, standard descriptive statistics such as the Coefficient of Variation (CV) or an analysis of the distribution skewness would be more biologically relevant and easier to interpret for the cell biology community. In addition, the interpretation of the LD number-cell size correlation as evidence for 'balanced biogenesis and inheritance' appears speculative in the absence of dynamic or lineage-resolved data

* Additionally, in Figure 6, Figure 7A, and Figure S9A the authors should provide a clear explanation of the violin plots, including the meaning of solid and dashed lines, the error lines, the symbols shown within the violins.

c. Were the data analyzed using appropriate statistical tests?

* yes

2. Reproducibility

a. Were experiments in each figure performed using adequate number of biological replicates?

* The authors state in the method section that all experiments were performed in three biological replicates unless otherwise stated in the text. However, I have some concerns regarding datasets presented in Figure 6:

* Figure 6A: Figure 6A reports only four data points for cell volume measurements obtained with the CASY™ system. This sample size is insufficient to support statistical conclusions.

* Figure 6B & Figure S8: The authors must clarify if the 'two dots' in the CTL quantification plot represent independent biological replicates. If these points correspond to the two different loading volumes (6 µl and 9 µl) of the same extract shown in Figure S8, they constitute technical replicates, which are statistically insufficient to support biological conclusions.

b. Is there sufficient raw data to assess the rigor of the analysis?

* The number of cells used for the quantitative analysis are sufficient

* However, the lack of clear information on the datasets indicated in the precedent paragraph raises some concerns

a. Does the methods section provide sufficient detail to permit reproducibility?

* The methods section is pretty detailed, provides sufficient information to allow technical reproducibility.

3. Completeness

a. Are the author's conclusions supported by the data?

* Overall, the workflow built by the authors systematically mitigate with efficiency critical sources of bias that can occur during analysis of LDs.

b. Are there any flaws in the experimental design that invalidate the approach taken by the authors?

* The approach taken by the authors is solid. However, as a methods-focused manuscript, the study would benefit from a broader benchmarking against state-of-the-art approaches. E.g., for cell segmentation the comparison is currently limited to global thresholding methods (Otsu and Huang), which represent legacy approaches. Comparison with machine-learning-based tools (e.g. Cellpose,

© 2026. Published by The Company of Biologists under the terms of the Creative Commons Attribution License (https://creativecommons.org/licenses/by/4.0/).

StarDist, or comparable yeast-optimized pipelines) would support the advance beyond the current state of the field or the better feasibility of their method.

c. Are there experiments that have not been performed, but if true would disprove the conclusion? If yes, and if such experiments would be costly or time-consuming to perform, do the authors acknowledge this in a discussion of the limitations?

* The pipeline is primarily validated using a mutant with extreme LD morphology. The authors acknowledge that no experiments are presented to demonstrate sensitivity to more subtle, physiologically relevant changes (e.g. different growth phases, nutrient conditions, or fatty acid supplementation), which would be important to establish the sensitivity and the broader applicability of the method.

4. Scholarship

a. Do the authors cite and discuss the merits of relevant data that would argue against their conclusion?

* The authors discuss the limitations of their approach

b. Do the authors cite and discuss the merits of relevant data that would support their conclusion?

* The authors discuss the merits of the data supporting their conclusion

c. For techniques/methods manuscripts, Do the authors cite and discuss the current state of the field and clearly explain how the method improves the field?

* The manuscript does not sufficiently discuss how the proposed pipeline improves upon existing high-throughput yeast LD analysis tools beyond classical thresholding approaches. A more comprehensive discussion of current deep-learning-based segmentation literature, including limitations and advantages relative to the present method, would strengthen the manuscript.

Additionally, there is a minor inconsistency in the supplementary material (e.g. references to Supplementary Figure 10 preceding Supplementary Figure 9); for legend clarity: the authors must specify the staining or fluorescent markers used in Figure 1. While the Methods section mentions BODIPY, the figure legend and labels fail to identify the signals for Lipid Droplets (LDs) and cells.

Reviewer's Responses to Questions

Experimental quality
Does each figure have the proper controls?
If 'No', please indicate reasons in Comments for Author box below.
Reviewer #1:
- Yes

Reviewer #2:
- No

Were the data analyzed using appropriate statistical tests?
If 'No', please indicate reasons in Comments for Author box below.
Reviewer #1:
- Yes

Reviewer #2:
- Yes

© 2026. Published by The Company of Biologists under the terms of the Creative Commons Attribution License (https://creativecommons.org/licenses/by/4.0/).

Reproducibility
Were experiments performed using adequate number of biological replicates?
If 'No', please indicate reasons in Comments for Author box below.
Reviewer #1:
- Yes

Reviewer #2:
- No

Does the methods section provide sufficient detail to permit reproducibility?
If 'No', please indicate reasons in Comments for Author box below.
Reviewer #1:
- Yes

Reviewer #2:
- Yes

Completeness
Are the manuscript's conclusions supported by the data?
If 'No', please indicate reasons in Comments for Author box below.
Reviewer #1:
- Yes

Reviewer #2:
- Yes

Scholarship
Do the authors cite and discuss the merits of data that would argue for and against their conclusion?
If 'No', please indicate reasons in Comments for Author box below.
Reviewer #1:
- Yes

Reviewer #2:
- Yes

Does the manuscript title & abstract accurately reflect the contents of the manuscript, without hyperbole?
If 'No', please indicate reasons in Comments for Author box below.
Reviewer #1:
- Yes

Reviewer #2:
- Yes

First revision

Author response to reviewers' comments

We thank the reviewers for their constructive comments. We revised the manuscript accordingly and thereby further improved our work. We included new features in selected modules, including support for CV, skewness, and CI95 metrics, and integrated a feature to process StarDist-generated labels within our statistical framework. In addition, we provide

© 2026. Published by The Company of Biologists under the terms of the Creative Commons Attribution License (https://creativecommons.org/licenses/by/4.0/).

new TLC data and have expanded the discussion section. Furthermore, we performed additional experiments to address specific reviewer questions, which are presented in the separately uploaded PDF entitled “Reviewer material.pdf”. In the point-by-point response below, the corresponding material is referenced as Figures R1–R5.

Comments from the Reviewers:

Reviewer 1: Summary

Gritsch et al describe a method that employs the use of radial raytracing and supervised machine learning to explore lipid droplets in yeast. Specifically, comparing wild type and mutant cells lacking the Sei1-Ldb16 seipin complex. The study is well thought out and easy to follow, with a clear and concise conclusions. This study will be a valuable contribution to the field, and I agree with the conclusion of the paper that "we see our approach as an experimental platform for community-driven refinement of LD quantification and related image-based analyses". While overall the manuscript is well written I have noted a few grammatical inaccuracies and areas that could improve impact of the manuscript.

Materials and Methods

1. Line 252 remove less than or equal to sign and replace with less than sign.

Answer: We replaced the “less than or equal” sign with the “less” sign.

Figures

1. To improve visualisation and interpretation, add trend lines to individual graphs of figure 7B and p-values for correlation analysis.

Answer: We tested the incorporation of linear trendlines for the scatter plots in our statistics script. However, Fig. R1 illustrates that these trendlines may be misleading. In the mutant, outliers in the LD area/cell area scatter plot can affect the fitted trendline, making it appear slightly more positive than in WT, i.e. the visual impression could suggest a stronger association in the mutant, although the corresponding Pearson correlation coefficients indicate a stronger correlation in WT. We therefore did not include automatic trendlines as a default feature of the statistics module, because they may promote overinterpretation of relationships particularly in yeast cell populations, where outliers in LD number/size and cell size can be expected.

*** Instead, to improve interpretability, we added the requested p-values directly to the plots in addition to the r-values. We also included CI95 values (95 % confidence intervals) where applicable. In addition, we indicated significance levels by stars in the plots. Together, we think that these additions provide a clearer statistical interpretation of the scatter plots without relying on potentially misleading trendlines. All corresponding values are reported in the spreadsheet output. We hope that this alternative adequately addresses the reviewer’s request for improved interpretability and visualization.

Furthermore, the purpose of these plots was to assess whether cell size is associated with LD size and number at the level of individual cells within the entire imaged population. Therefore, for this specific analysis, the values from the three replicates were initially pooled (r-values), whereas all other metrics were calculated from replicate means.

*** For consistency with the other metrics, we now calculated Pearson correlations separately for each biological replicate and summarized the replicate-specific r-values using Fisher’s transformation method. We adapted the statistics module and the graphs accordingly (the resulting r-values were not substantially changed). When the grouping feature is selected, correlation metrics are computed replicate-wise; if this feature is not

© 2026. Published by The Company of Biologists under the terms of the Creative Commons Attribution License (https://creativecommons.org/licenses/by/4.0/).

used, values are calculated from pooled data.

Further to this, p-values should be added in the text next to the values they relate to, for example, on line 417 a r-value of -0.81 is in the brackets only.

Answer: We included the relevant p-values in the text addressing the correlation plots.

Discussion

1. In the literature do you see in sei1Δ ldb16Δ double deletion mutant cells a similar magnitude of effect when compared to your model partnered with your analysis technique? Are there comparable LD analyses that you could cite as a direct comparator

Answer: We did not find studies of the double deletion mutant in 48 h culture in rich medium and using optimized high-resolution imaging as in our manuscript. Most studies used log-phase or "overnight cultures" under synthetic complete medium conditions (with inositol or without inositol). In addition, studies of the double deletion mutant are rare. Most studies analyzed the single deletion mutants (*sei1Δ* or *ldb16Δ* cells).

Regarding the observed shift towards very small LDs <200 nm and large LDs/LD clusters in the seipin mutant: in the original yeast seipin paper, Fei et al. 2008. JCB 180,473-482, they found in fld1Δ (sei1Δ) cells a mixed population of clustered and supersized LDs together with small LDs in overnight cultures in rich medium. Using electron microscopy and in line with our observations in the double deletion mutant, they also showed that the cells can contain a large number of very small LDs of < 0.2 µm, which were distributed throughout the cell (Figure 1B h-i in this paper). These findings are consistent with our observations.

*** We had already cited the Fei et al. paper in this context but have now extended the text for further clarification including the previous TEM observations.

Renne et al. (Renne, et al. 2022. *J. Cell Biol.* 221; Fig. 3A-B, top row) also observed a clear shift of LD size toward smaller and very small LDs together with a few enlarged LDs in the single mutants and the double deletion mutant under SC-ino conditions, whereas they used a high-speed "super-resolution" imaging method for image acquisition.

*** Since these observations are also in line with our results, we additionally cited this paper, while noting the different growth medium conditions.

Regarding the correlation of LD size and LD number with cell size in WT cells: to our best knowledge, studies on the correlation of LD size and cell size in both WT and seipin mutants have not been carried out in S. cerevisiae so far. We found a study in an oleaginous yeast showing by FACS that the fluorescence intensity of Nile Red-labeled LDs indicating enlargement of the organelles increased together with the cell size of this fungus, which may support our findings in S. cerevisiae (Raschke and Knorr. 2009. Journal of Microbiological Methods 79(2):178-183.).

*** Although the experiments were performed in different yeast species, we now cite this paper accordingly.

Regarding the observed cell-to-cell heterogeneity of the occurrence of supersized/clustered LDs in the double deletion mutant:

In a previous work, we already observed a striking heterogeneity of the occurrence of the LD phenotypes in maximum-intensity projections of z-stacks acquired from the double deletion mutant under SC+ino conditions but also in the single mutants under SC +/- ino conditions (16h cultures): Small LDs in a fraction of cells may represent developing clusters (Wolinski et al. 2015. BBA Mol Cell Lipids 1851(11),1450-1464; Fig. S1, bottom panel). Seipin

© 2026. Published by The Company of Biologists under the terms of the Creative Commons Attribution License (https://creativecommons.org/licenses/by/4.0/).

mutants show a partial defect in LD inheritance (Wolinski et al., 2011. JCS 124, 3894-3904), which may affect the LD phenotype particularly of the last cell generation, thereby contributing to cell-to-cell heterogeneity in stationary-phase cells. In addition, seipin LD phenotypes depend strongly on cultivation and medium conditions; for example, the yeast extract used for YPD rich medium is not standardized which may also affect the detailed LD distribution in the cell population.

*** We have addressed this point briefly in the discussion section.

2. Line 558 authors suggest an alternative to BODIPY 493/503 staining. I wonder if whether it's worth mentioning that fluorescently labelled fusion protein that are LD specific could also be used as an alternative especially to explore nascent LDs that may be poorly detected with BODIPY 493/503.

Answer: The cited Wang et al. 2016 paper describes the LiveDrop fusion marker for detection of early LDs.

*** We now explicitly mention LiveDrop as a fusion-protein marker for nascent LDs.

A few minor suggestions to improve readability

1. Line 554 remove "also"

Answer: Removed.

2. Line 608 minor grammatical error I believe it should read "be most active at ER fractions close to the nuclear envelope..."

Answer: In our previous work (Wolinski et al. 2015. BBA Mol Cell Lipids 1851(11),1450-1464), we proposed that there is a subdomain or ER fraction at the nuclear envelope where LDs may preferentially accumulate ("LD assembly domain").

*** In the context of this work, we have now used the more general wording: "… active at an ER fraction …".

3. Line 635 I would remove the words "poorly useful" and replace with suboptimal

Answer: We replaced this term accordingly with "suboptimal."

Reviewer 2: 1. Experimental quality

a. Does each figure have the proper controls?

*** In Figure 5 the authors assign LD to yeast cells, but they don't show LD: the reader cannot visually verify whether the assignment of LDs to segmented cells is accurate. Overlaying the LD channel with the transmission image, or providing zoomed-in insets showing LD localization, would greatly improve the interpretability of this figure.**

Answer: This is correct. This image shows the assignment of the lipid droplet size classes as colored symbols generated by the LD_CellAssigner script rather than fluorescently labeled LDs.

*** We included an additional inset showing the fluorescence/SRR overlay output in Fig. 5. The segmentation data of all images are provided in the */descriptor/overlays folder.

© 2026. Published by The Company of Biologists under the terms of the Creative Commons Attribution License (https://creativecommons.org/licenses/by/4.0/).

b. Are experiments performed using appropriate methods that will answer the question (or test the hypothesis or support the observations) posed by the authors? Is the right tool used for the job?

*** Overall, the imaging and analysis approaches are appropriate for the authors' stated goals.**

*** In Figure 7A the use of Shannon and Pielou indices is unconventional for organelle distribution analysis (and strongly dependent on binning choices). While creative, the authors should justify why these ecological metrics are superior to standard distribution descriptors (e.g., CV or Skewness) for LD populations.**

To describe LD heterogeneity, standard descriptive statistics such as the Coefficient of Variation (CV) or an analysis of the distribution skewness would be more biologically relevant and easier to interpret for the cell biology community.

Answer: We thank the reviewer for this comment. We agree that Shannon and Pielou indices are less commonly used for LD analysis than CV or skewness, and that their use therefore requires additional explanation. Our aim was not to describe the spread of LD sizes around a mean value, for which CV and skewness can be useful, but to describe how LDs are distributed across our predefined size classes. For this purpose, Shannon and Pielou indices provide useful information. Shannon entropy reflects the diversity of LD size-class occupancy, whereas Pielou evenness indicates how evenly LDs are distributed across these classes. These parameters therefore help to distinguish whether an LD population is dominated by one size class or whether LDs are distributed more broadly and evenly across the size spectrum. In addition, the violin plots further show how Shannon and Pielou values vary across individual cells. In contrast, CV and skewness describe the relative dispersion and asymmetry of LD sizes but do not specify how LDs are distributed among the predefined size classes, which is the focus of our approach.

*** In this context, we agree that CV and skewness are important descriptors of LD-size distributions. We have therefore included a feature in the statistics module and added both metrics to the output of the statistics script (merged_stats) as additional output, whereas CV and skewness were also calculated within the same size-class framework and based on replicate-means.

As expected, the mutant showed a higher CV of LD size than WT cells of ca. 46 % versus ca. 27 % and stronger positive skewness of 1.28 versus 0.25, indicating increased relative dispersion and a right-tailed (larger) LD-size distribution (the values derive from the new spreadsheet output). However, a few large or supersized LDs in the population can strongly influence CV and skewness, while the dominant LD population still consists of small LDs as suggested by the size class analysis.

In addition, the interpretation of the LD number-cell size correlation as evidence for 'balanced biogenesis and inheritance' appears speculative in the absence of dynamic or lineage-resolved data.

Answer: We agree; this conclusion is speculative.

*** We corrected this phrase accordingly: “In addition, the relatively low variability within each cell-size profile may indicate balanced LD biogenesis and inheritance during growth. However, this remains speculative without dynamic or lineage-resolved data”

*** Additionally, in Figure 6, Figure 7A, and Figure S9A the authors should provide a clear explanation of the violin plots, including the meaning of solid and dashed lines, the error lines, the symbols shown within the violins.**

Answer: We now provide an explanation of the violin plots in the materials and methods section as well as in the figure legends.

© 2026. Published by The Company of Biologists under the terms of the Creative Commons Attribution License (https://creativecommons.org/licenses/by/4.0/).

c. Were the data analyzed using appropriate statistical tests?

* yes

2. Reproducibility

a. Were experiments in each figure performed using adequate number of biological replicates?

*** The authors state in the method section that all experiments were performed in three biological replicates unless otherwise stated in the text. However, I have some concerns regarding datasets presented in Figure 6:**

*** Figure 6A: Figure 6A reports only four data points for cell volume measurements obtained with the CASY™ system. This sample size is insufficient to support statistical conclusions.**

Answer: These data points represent mean cell-size values obtained with the CASY cell counter system, a standard device for cell-size measurement in yeast. The CASY system counts cells as they pass through an electro-sensing capillary during each measurement, typically analysing thousands of cells per run. The system then provides the mean cell size of the measured cell population. Thus, the plotted symbols do not represent individual cells, but independent experimental measurements/biological replicates.

*** We clarified this point in the legend text and added a description of the CASY measurement to the Materials and Methods section.

*** Figure 6B & Figure S8: The authors must clarify if the 'two dots' in the CTL quantification plot represent independent biological replicates. If these points correspond to the two different loading volumes (6 µl and 9 µl) of the same extract shown in Figure S8, they constitute technical replicates, which are statistically insufficient to support biological conclusions.**

Answer: This is correct. The values shown are technical replicates and serve as a raw reference for NL levels in these strains. The experiment was performed to show that image-based quantification of LD size is not a reliable measure of the NL content of cells, due to the still-present limitations of quantitative image analysis (e.g. different values when calculated per cell or per cell area; unclear effect of different neutral lipid packaging in a LD population), as discussed in the manuscript.

We carefully performed additional TLC experiments with three biological replicates. In these new experiments, no significant differences in NL levels between the WT and mutant strains were detected when normalizing to ergosterol levels, in contrast to the ~14% increase observed in the initial experiment.

This difference from the initial experiments is, however, not surprising. Measurements of TAG and/or SE content in seipin mutants have yielded divergent results so far. A slight increase in SE levels only, but not in TAG levels, was reported for the single-deletion mutants (*sei1Δ*, *ldb16Δ*) grown overnight in YPD to stationary phase (Wang et al., 2014. JCS 127(6):1214-1228; Fig. S1A). In contrast, Fei et al. (Fei et al. 2008. JCB 180,473-482) reported an approximately two-fold increase in TAG and SE levels in the SEI1 deletion mutant strain during log-phase and stated that similar results were also observed in stationary-phase YPD cultures, i.e. under nominally comparable cultivation conditions.

In this context, seipin LD phenotypes depend strongly on cultivation and media conditions; for example, the yeast extract used for YPD (rich medium) is not standardized. Furthermore, TLC is sensitive to experimental conditions, including chamber saturation, humidity, sample application etc. Therefore, samples analyzed within the same run are most directly comparable, whereas some variability between independent TLC runs cannot be fully excluded.

© 2026. Published by The Company of Biologists under the terms of the Creative Commons Attribution License (https://creativecommons.org/licenses/by/4.0/).

*** We adapted the results and discussion sections with the new data and emphasized more clearly the limitations of image-based quantification of NL content, which was the aim of the experiment, but also the semi-quantitative nature of TLC including a reference. In addition, we updated Fig. 6B and Fig. S8, and the legend text accordingly.

b. Is there sufficient raw data to assess the rigor of the analysis?

*** The number of cells used for the quantitative analysis are sufficient**

*** However, the lack of clear information on the datasets indicated in the precedent paragraph raises some concerns**

Answer: This concern is addressed by the additional TLC experiments described above and by clarification of the CASY replicates.

a. Does the methods section provide sufficient detail to permit reproducibility?

* The methods section is pretty detailed, provides sufficient information to allow technical reproducibility.

3. Completeness

a. Are the author's conclusions supported by the data?

* Overall, the workflow built by the authors systematically mitigate with efficiency critical sources of bias that can occur during analysis of LDs.

b. Are there any flaws in the experimental design that invalidate the approach taken by the authors?

*** The approach taken by the authors is solid. However, as a methods-focused manuscript, the study would benefit from a broader benchmarking against state-of- the-art approaches. E.g., for cell segmentation the comparison is currently limited to global thresholding methods (Otsu and Huang), which represent legacy approaches. Comparison with machine-learning-based tools (e.g. Cellpose, StarDist, or comparable yeast-optimized pipelines) would support the advance beyond the current state of the field or the better feasibility of their method.**

Answer (Otsu, Huang): We selected traditional thresholding methods because they are implemented in several widely used image-analysis and segmentation tools, including CellProfiler. The standard primary-object detection workflow in this tool relies largely on histogram- and threshold-based segmentation strategies including the Otsu method.

However, we prefer to avoid explicitly naming selected tools in this context, because we do not intend to question their general applicability, which can be highly beneficial for other imaging tasks.

*** We have now included a phrase in the discussion section stating that such and related methods are implemented in existing image-analysis tools.

Answer (StarDist): Benchmarking against other methods, particularly deep-learning approaches such as StarDist, is not straightforward, because their output depends not only on the training data (in the case of StarDist, nuclei) and the specific images, but also on model choice and, in particular on user-defined settings; i.e. the user can affect the results. The challenges of benchmarking in this context are discussed e.g. by Segebarth et al. 2020. eLife 9: e59780.

Nevertheless, we investigated the performance of the deep learning-based StarDist method for the detection of LDs in our already optimized LD images and compared the results with

© 2026. Published by The Company of Biologists under the terms of the Creative Commons Attribution License (https://creativecommons.org/licenses/by/4.0/).

those obtained by the SRR approach. We found that StarDist, although trained on nuclei, separated closely associated LDs better than conventional histogram- or threshold-based segmentation methods. However, the overall LD sizes appeared clearly overestimated compared to the SRR method. Small and dim LDs were partly not detected, and when they were detected, their sizes appeared to be significantly overestimated. In addition, closely associated and substantially overlapping LDs were not registered as individual objects under the default settings (Reviewer material.pdf; Fig. R2).

Furthermore, in contrast to the SRR-based analysis, StarDist did not detect a significant increase in mean LD count in *sei1Δ ldb16Δ* cells compared with WT, although the microscopy images and published data suggest an increase of the number of small LDs in the mutant. In this respect, under the tested default settings StarDist underestimated the contribution of small and closely associated LDs. Furthermore, the deep-learning tool showed substantially higher absolute LD area/cell values than SRR in both WT and mutant cells, but a smaller relative increase between WT and mutant. Thus, compared with SRR, StarDist reduces the apparent strength of the genotype-dependent shift in LD area per cell (Reviewer material.pdf; Fig. R3A).

Moreover, the LD size-class distribution computed using StarDist differed from the SRR output. In the mutant, the small LD class (<0.2 µm) was almost absent in the StarDist analysis, simply because this method could not segment these LDs. In SRR, the mutant-associated shift prominently involves the smallest LD class and an increased contribution of the largest class, whereas StarDist largely lacks the smallest fraction and instead shows a different relative redistribution among medium-to-large LD classes, most likely by artificial fusion of closely associated LDs in the segmented images (Reviewer material.pdf; Fig. R3B).

We would like to point out that our work is not primarily intended to provide just another software tool for LD quantification. Rather, SRR uses a PSF- and related intensity-profile-informed detection strategy and therefore represents a fundamentally different conceptual approach from histogram-based and deep-learning-based segmentation methods. Because it is grounded in imaging physics, this framework offers opportunities for systematic refinement beyond empirical parameter tuning.

In particular, using this method, we aim to move toward “reference-based, calibrated” quantification of LD sizes. In this context, the simple SRR approach can be further improved and calibrated using synthetic or hybrid synthetic-experimental image datasets that reproduce not only optical properties such as PSF-related blurring and noise, but also experiment-inherent features, including slight LD motion, ambiguous signals between closely spaced droplets, as well as biologically induced variation in intensity decay profiles. We have already initiated experiments in this direction; however, these experiments are still in a preliminary phase and therefore cannot be used for this manuscript.

*** Since the evaluation of benchmark results is limited, we did not include these experiments in the manuscript. Alternatively, we included a component into the LD_Segmenter_v1.py module (renamed LD_HistogramSegmenter_v1.py module) for processing of StarDist labels. This feature allows StarDist labels generated in Fiji to be imported, assigned to cell masks, and processed for LD statistics, including size-class distribution (as shown in Fig. R3B, Reviewer material.pdf), thereby extending our experimental platform with this feature. In addition, we adapted the help file for this module accordingly.

*** We now address these aspects in the discussion section including the route towards “calibration” standards for LD size determination.

Answer (CellPose): Furthermore, YeastSpotter was used for cell (boundary) detection in our workflow. As indicated in the flow chart in Fig. S1 of the manuscript, any alternative method, e.g. cell binary images generated by CellPose (Stringer, et al. 2021. *Nature Methods* 18, 100-106.), can be used. In our transmission sum projections, however, this approach primarily detects the outer cell border, whereas the YeastSpotter method in our setup primarily detects contrast associated with the cell interior, while the outer cell boundary is represented less consistently. Since the apparent thickness of this boundary (cell wall)

© 2026. Published by The Company of Biologists under the terms of the Creative Commons Attribution License (https://creativecommons.org/licenses/by/4.0/).

depends on the optical configuration of the light microscope, for example the condenser position, we preferred the YeastSpotter setup for our specific application.

*** We included a note in the results section that other cell segmentation techniques such as CellPose can be used as well.

c. Are there experiments that have not been performed, but if true would disprove the conclusion? If yes, and if such experiments would be costly or time-consuming to perform, do the authors acknowledge this in a discussion of the limitations?

*** The pipeline is primarily validated using a mutant with extreme LD morphology. The authors acknowledge that no experiments are presented to demonstrate sensitivity to more subtle, physiologically relevant changes (e.g. different growth phases, nutrient conditions, or fatty acid supplementation), which would be important to establish the sensitivity and the broader applicability of the method.**

Answer: We performed further morphometric analyses of LDs in WT cells under lipolytic and fatty-acid induced LD formation conditions. In summary, the SRR-based quantifications reproduced the expected organelle remodelling during lipolysis and under conditions of stimulated LD synthesis, again indicating the robust basic performance of the SRR approach.

Consistent with previous works, WT cells re-inoculated into fresh medium to stimulate cellular growth and lipolysis (Athenstaedt, K. and Daum, G. 2003. J. Biol. Chem. 278, 23317–23323; Kurat et al. J. Biol. Chem. 281, 491–500) showed a reduced LD number and size. In contrast, as expected oleate-treated cells displayed increased LD area compared with untreated cells due to the stimulated synthesis of NLs (Grillitsch et al. BBA Molecular and Cell Biology of Lipids 1811, 1165–1176; Reviewer material.pdf; Fig. R4).

However, the analysis also revealed more subtle differences. Compared with values obtained from 48 h cultures (Fig. 6B manuscript), oleate-treated cells showed only a slightly higher LD number per cell (oleate treated cells 5.466; 48h cultures, ~5.0866 LDs/cell). This result may indicate that a substantial fraction of oleate is incorporated into pre-existing LDs. In addition, fatty acid supplemented cells showed a still reduced total LD area per cell compared to 48 h cultures (48 h cultures: ~0.26 µm2 / cell (Fig. 6B; manuscript); oleate treated cells: ~0.22 µm2 per cell). We speculate that, at least during the initial phase after re-inoculation, lipolysis may still continue even under oleate- supplemented conditions, while oleate shifts the overall balance toward NL synthesis (Reviewer material.pdf; Fig. R4).

Furthermore, the correlation between LD number and cell size was relatively maintained under both conditions (treated: r ~ 0.74; untreated: r ~ 0.71). However, the correlation between LD area and cell area was lower in oleate-treated cells (r ~ 0.60), compared with untreated cells (r ~ 0.69) and 48 h cultures (r ~ 0.71) (Reviewer material.pdf; Fig. R5A). Shannon and Pielou indices further indicated that fatty acid treatment led to a more heterogeneous distribution across the predefined LD-size classes than in untreated cells, although this heterogeneity remained lower than in stationary-phase cultures. This may suggest that oleate is incorporated into the existing LD pool less uniformly than LD formation under de novo synthesis conditions (Reviewer material.pdf; Fig. R5B).

We did not include these additional experiments in this manuscript but provide the results in the provided Reviewer material.pdf.

4. Scholarship

a. Do the authors cite and discuss the merits of relevant data that would argue against their conclusion?

* The authors discuss the limitations of their approach

b. Do the authors cite and discuss the merits of relevant data that would support their conclusion?

© 2026. Published by The Company of Biologists under the terms of the Creative Commons Attribution License (https://creativecommons.org/licenses/by/4.0/).

* The authors discuss the merits of the data supporting their conclusion

c. For techniques/methods manuscripts, Do the authors cite and discuss the current state of the field and clearly explain how the method improves the field?

*** The manuscript does not sufficiently discuss how the proposed pipeline improves upon existing high-throughput yeast LD analysis tools beyond classical thresholding approaches. A more comprehensive discussion of current deep-learning-based segmentation literature, including limitations and advantages relative to the present method, would strengthen the manuscript.**

Answer: We now discuss the differences between existing deep-learning methods such as StarDist and the SRR method in the discussion section. We have shown a "benchmark" between StarDist and SRR in the response to question 3b.

Additionally, there is a minor inconsistency in the supplementary material (e.g. references to Supplementary Figure 10 preceding Supplementary Figure 9); for legend clarity: the authors must specify the staining or fluorescent markers used in Figure 1.

While the Methods section mentions BODIPY, the figure legend and labels fail to identify the signals for Lipid Droplets (LDs) and cells.

Answer: We added a corresponding label in Fig. 1 for clarification. In addition, we added a short explanatory text to the figure legends of images showing BODIPY 493/503-labeled LDs, where applicable.

Second decision letter

MS ID#: bio.062429R1

MS Title: Lipid droplet size profiling in yeast

Authors: Katharina Gritsch; Klara Skrobar Martincic; Kristian Bredies; Heimo Wolinski

I have now reached a decision on the above manuscript.

The reviewer reports are shown at the bottom of this email. As you will see, the reviewers gave very favourable reports. I normally would be accepting your manuscript, but in light of your much appreciated transparency about having to repeat some experiments for Figure 6, I want to give you the opportunity to replace Figure 6, make any changes in the figure legend required, and add new supplementary material. Having looked over the data you sent, the conclusions of the manuscript are not changed, but I just want to be sure that you have the opportunity to correctly include the new data in your manuscript.

Reviewer 1

Comments for the author

Reading through the author's response to reviewer comments I believe all questions have been answered satisfactory. Likewise, the revised manuscript reads much more clearly and I believe has far more impact.

Reviewer 2

© 2026. Published by The Company of Biologists under the terms of the Creative Commons Attribution License (https://creativecommons.org/licenses/by/4.0/).

Comments for the author

I would like to thank the authors for their detailed point-by-point response, the clarifications, and for performing new evidences to support their main claims. All my previous concerns have been addressed satisfactorily.

Reviewer's Responses to Questions

Experimental quality
Are experiments performed using appropriate methods that will answer the question (or test the hypothesis or support the observations) posed by the authors? Is the right tool used for the job?
If 'No', please indicate reasons in Comments for Author box below.
Reviewer #1:
- Yes

Reviewer #2:
- Yes

Were the data analyzed using appropriate statistical tests?
If 'No', please indicate reasons in Comments for Author box below.
Reviewer #1:
- Yes

Reviewer #2:
- Yes

Reproducibility
Were experiments performed using adequate number of biological replicates?
If 'No', please indicate reasons in Comments for Author box below.
Reviewer #1:
- Yes

Reviewer #2:
- Yes

Is there sufficient raw data to assess the rigor of the analysis?
If 'No', please indicate reasons in Comments for Author box below.
Reviewer #1:
- Yes

Reviewer #2:
- Yes

Does the methods section provide sufficient detail to permit reproducibility?
If 'No', please indicate reasons in Comments for Author box below.
Reviewer #1:
- Yes

Reviewer #2:
- Yes

Completeness

© 2026. Published by The Company of Biologists under the terms of the Creative Commons Attribution License (https://creativecommons.org/licenses/by/4.0/).

Are the manuscript's conclusions supported by the data?
If 'No', please indicate reasons in Comments for Author box below.
Reviewer #1:
- Yes

Reviewer #2:
- Yes

Scholarship
Do the authors cite and discuss the merits of data that would argue for and against their conclusion?
If 'No', please indicate reasons in Comments for Author box below.
Reviewer #1:
- Yes

Reviewer #2:
- Yes

Does the manuscript title & abstract accurately reflect the contents of the manuscript, without hyperbole?
If 'No', please indicate reasons in Comments for Author box below.
Reviewer #1:
- Yes

Reviewer #2:
- Yes

Second revision

Author response to reviewers' comments

We revised the manuscript according to the constructive suggestions of the reviewers and were thereby able to further improve our work. We included new statistical metrics (CV, skewness, CI95) and functionality to process imported StarDist labels within our statistical framework, included new data from thin-layer chromatography, added annotations to the graphs, and expanded the discussion section as requested. In addition, we performed further experiments to address specific reviewer questions. In addition, we briefly outline future reference-based calibration of SRR-based LD-size quantification using synthetic hybrid datasets that model optical effects and variation of acquired images.

Third decision letter

MS ID#: bio.062429R2

MS Title: Lipid droplet size profiling in yeast

Authors: Katharina Gritsch; Klara Skrobar Martincic; Kristian Bredies; Heimo Wolinski

I am happy to tell you that your manuscript has been accepted for publication in Biology Open, pending our standard publication integrity checks. It was accepted on 9th June 2026.

© 2026. Published by The Company of Biologists under the terms of the Creative Commons Attribution License (https://creativecommons.org/licenses/by/4.0/).

Reviewer 1

Comments for the author

This revision was warranted to assess the updated figure as indicated by the corresponding author. I am satisfied with the updated figure 6A and have reviewed the corresponding raw data and believe no further changes are required.

Reviewer 2

Comments for the author

I would like to thank the authors for their detailed responses and for their thorough revision of the manuscript. Furthermore, I would like to commend them for their honesty and scientific integrity.

All my initial concerns have been fully addressed, and since the main conclusions remain completely unchanged and the authors have updated the manuscript accordingly, I am happy to recommend this manuscript for publication.

Reviewer's Responses to Questions

Experimental quality
Are experiments performed using appropriate methods that will answer the question (or test the hypothesis or support the observations) posed by the authors? Is the right tool used for the job?
If 'No', please indicate reasons in Comments for Author box below.
Reviewer #1:
- Yes

Reviewer #2:
- Yes

Were the data analyzed using appropriate statistical tests?
If 'No', please indicate reasons in Comments for Author box below.
Reviewer #1:
- Yes

Reviewer #2:
- Yes

Reproducibility
Were experiments performed using adequate number of biological replicates?
If 'No', please indicate reasons in Comments for Author box below.
Reviewer #1:
- Yes

Reviewer #2:
- Yes

Is there sufficient raw data to assess the rigor of the analysis?
If 'No', please indicate reasons in Comments for Author box below.
Reviewer #1:
- Yes

Reviewer #2:

© 2026. Published by The Company of Biologists under the terms of the Creative Commons Attribution License (https://creativecommons.org/licenses/by/4.0/).

- Yes

Does the methods section provide sufficient detail to permit reproducibility?
If 'No', please indicate reasons in Comments for Author box below.
Reviewer #1:
- Yes

Reviewer #2:
- Yes

Completeness
Are the manuscript's conclusions supported by the data?
If 'No', please indicate reasons in Comments for Author box below.
Reviewer #1:
- Yes

Reviewer #2:
- Yes

Scholarship
Do the authors cite and discuss the merits of data that would argue for and against their conclusion?
If 'No', please indicate reasons in Comments for Author box below.
Reviewer #1:
- Yes

Reviewer #2:
- Yes

Does the manuscript title & abstract accurately reflect the contents of the manuscript, without hyperbole?
If 'No', please indicate reasons in Comments for Author box below.
Reviewer #1:
- Yes

Reviewer #2:
- Yes

© 2026. Published by The Company of Biologists under the terms of the Creative Commons Attribution License (https://creativecommons.org/licenses/by/4.0/).
